# Highly Sensitive Virome Characterization of *Aedes aegypti* and *Culex pipiens* Complex from Central Europe and the Caribbean Reveals Potential for Interspecies Viral Transmission

**DOI:** 10.3390/pathogens9090686

**Published:** 2020-08-21

**Authors:** Jakob Thannesberger, Nicolas Rascovan, Anna Eisenmann, Ingeborg Klymiuk, Carina Zittra, Hans-Peter Fuehrer, Thea Scantlebury-Manning, Marquita Gittens-St.Hilaire, Shane Austin, Robert Clive Landis, Christoph Steininger

**Affiliations:** 1 Division of Infectious Diseases, Department of Medicine 1, Medical University of Vienna, 1090 Vienna, Austria; jakob.thannesberger@meduniwien.ac.at (J.T.); anna.eisenmann@gmx.at (A.E.); 2 Department of Genomes & Genetics, Institut Pasteur, 75015 Paris, France; nicorasco@gmail.com; 3 Center for Medical Research, Core Facility Molecular Biology, Medical University of Graz, 8036 Graz, Austria; ingeborg.klymiuk@medunigraz.at; 4 Institute of Parasitology, University of Veterinary Medicine, 1210 Vienna, Austria; carina.zittra@univie.ac.at (C.Z.); Hans-Peter.Fuehrer@vetmeduni.ac.at (H.-P.F.); 5 Unit Limnology, Department of Functional and Evolutionary Ecology, University of Vienna, 1010 Vienna, Austria; 6 Department of Biological and Chemical Sciences, Faculty of Science and Technology, Cave Hill Campus, The University of the West Indies, Bridgetown BB11000, Barbados; thea.scantlebury-manning@cavehill.uwi.edu (T.S.-M.); shane.austin@cavehill.uwi.edu (S.A.); 7 Faculty of Medical Sciences, University of the West Indies, Queen Elizabeth Hospital, St. Michael BB14004, Barbados; marquita.gittens@cavehill.uwi.edu; 8 Edmund Cohen Laboratory for Vascular Research, George Alleyne Chronic Disease Research Centre, The University of the West Indies, Bridgetown BB11115, Barbados; clive.landis@cavehill.uwi.edu

**Keywords:** mosquito virome, CRESS-DNA viruses, CyCV-VN, insect-specific viruses, ISV, BatCV

## Abstract

Mosquitoes are the most important vectors for arthropod-borne viral diseases. Mixed viral infections of mosquitoes allow genetic recombination or reassortment of diverse viruses, turning mosquitoes into potential virologic mixing bowls. In this study, we field-collected mosquitoes of different species (*Aedes aegypti* and *Culex pipiens complex*), from different geographic locations and environments (central Europe and the Caribbean) for highly sensitive next-generation sequencing-based virome characterization. We found a rich virus community associated with a great diversity of host species. Among those, we detected a large diversity of novel virus sequences that we could predominately assign to circular Rep-encoding single-stranded (CRESS) DNA viruses, including the full-length genome of a yet undescribed *Gemykrogvirus* species. Moreover, we report for the first time the detection of a potentially zoonotic CRESS-DNA virus (*Cyclovirus VN)* in mosquito vectors. This study expands the knowledge on virus diversity in medically important mosquito vectors, especially for CRESS-DNA viruses that have previously been shown to easily recombine and jump the species barrier.

## 1. Introduction

Mosquitoes represent the most medically important group of arthropod vectors. In particular, mosquitoes of the *Aedes* and *Culex* genera are well adapted to human environments, making them the most important transmission vectors for arthropod-borne viruses (arboviruses) such as Dengue virus, Zika virus, or West Nile virus [1]. Nevertheless, arboviruses sensu stricto are not the only viruses that replicate in mosquito vectors [2,3,4,5].

Mosquitoes may be infected by a range of insect-specific viruses (ISVs) that replicate only in arthropod cells, unlike arboviruses that also replicate in vertebrate cells. Arboviruses are closely related to ISVs in terms of biological properties and phylogenetic distance and may have arisen from ISVs that occasionally gained the capacity to infect secondary hosts [6,7,8]. Most RNA ISVs are classified in the families of *Flaviviridae*, *Togaviridae*, the order of *Bunyavirales*, and, recently, *Mesoniviridae* [6]. Despite being confined to the arthropod host, ISVs are medically relevant as they may alter arbovirus replication and transmission [6,9,10,11,12]. For example, coinfection with ISVs has been shown to attenuate arbovirus replication in *Aedes* cell lines in vitro, including West Nile virus, Japanese encephalitis virus, and Zika virus [13,14]. In vivo studies have further confirmed that dissemination of the West Nile virus is attenuated in naturally ISV-infected mosquito populations [15].

After identification of the first ISV (cell-fusing agent virus) in 1975, it took almost 25 years for the second ISV to be discovered (Kamiti river virus) and seven more years for the third (Culex flavivirus) [6]. While advances in metagenomic sequencing are constantly accelerating the detection rate of novel ISVs, the vast diversity of ISVs has yet to be fully explored and understood [6]. Comprehensive knowledge of ISV diversity and abundance is pivotal to understanding arbovirus evolution and the interaction of ISVs with medically important mosquito vectors. Thereby, it may also help to identify ways to use ISVs in arbovirus control efforts. 

In addition to arboviruses and ISVs, mosquitoes may temporarily host additional virus species that do not replicate in the arthropod hosts but become transiently associated with the mosquito vector through dynamic interaction with virus sources of the prevailing environment. For example, depending on species and sex, mosquitoes can be nourished on the blood of vertebrate hosts (female mosquitoes only) or on the nectar of pollinating plants (both female and male). The diversity of the mosquito virome is, therefore, in constant flux, depending on the surrounding environment [4]. Viruses ingested from other host organisms may, therefore, constitute an abundant and diverse fraction of the mosquito virome, even if they may not be capable of replicating within the mosquito [16].

The presence of a range of viruses from different hosts may drive interspecies recombination or reassortment within mosquitoes and promote the emergence of novel virus species [17]. The likelihood of a recombination event is increased when phylogenetically closely related viruses are present in the same biological reservoir, and this may be especially true of viruses with low-complexity genomes such as circular Rep-encoding ssDNA viruses (CRESS-DNA viruses) [18]. CRESS-DNA viruses undergo rapid evolution and are prone to recombination events due to their highly conserved genome organization throughout all known lineages. Genomic analysis of the recently identified CRESS-DNA virus Porcine circovirus 3 revealed that the virus genome was most likely formed by recombination of avian and mammalian (especially bat) Circovirus strains, which subsequently led to a species jump into pigs and an attendant economic loss of livestock production [19]. Mixed CRESS-DNA virus infections of mosquito vectors might, therefore, drive virus recombination events and subsequent formation of novel viruses with an altered host spectrum. CRESS-DNA viruses are highly abundant in many ecosystems and have recently gained attention as potential human pathogenic viruses [18].

The replication of arboviruses and ISVs within the same biological compartment and the transfer of viruses between ecological niches provide the prerequisite for the emergence of novel mutant viruses with pathogenic potential. Metagenomic analysis of the mosquito virome can promote unbiased detection of viruses that might not have been associated with mosquito vectors before, including zoonotic viruses. In this study, we aim to analyze the metagenomic virome diversity of medically important mosquito vectors. We performed highly sensitive virus metagenome sequencing of field-collected *Aedes aegypti* (*Ae.ae*.) and *Culex pipiens* complex (*C.pip.cl*.) mosquitoes from the Caribbean (Barbados; *Ae. aegypti* and *Cx. pipiens* complex) and central Europe (Austria; *Cx. pipiens* complex) to conduct comparative virome analysis of the two ecologically divergent areas. We characterized phylogenetic virome patterns and the spectrum of virus-associated hosts. Viral hits were categorized by concordance with database entries, hence revealing any novel viruses. Among those, we describe the genome organization of a novel virus of the genus of *Gemykrogvirus* and, for the first time, identify a potentially zoonotic Cyclovirus (*CyCV-VN*) in mosquito vectors.

## 2. Material and Methods

### 2.1. Mosquito Collection and Taxonomic Identification

Mosquito collection was performed as previously described, using carbon dioxide-equipped BG sentinel traps (Biogents AG, Regensburg, Germany) for 24-h time periods [20]. To compare sites of divergent ecological preconditions, mosquitoes were collected in Austria and Barbados. Austria is located in the temperate humid region of central Europe. Barbados, a Caribbean island of the West Indies, is within the tropical humid region.

In Austria, traps were set up at two trapping sites at the municipal area of Vienna (Supplementary Table S1). Locations were characterized based on satellite images (Google^®^ Inc, Mountain View, CA, USA) and land cover maps provided by the Austrian Environment Agency (CORINE Land cover map Austria; Supplementary Figure S4) [21]. Calculations on absolute and relative coverage were done in ImageJ [22]. Mosquito collection in Barbados was performed during October and November 2016 over eight consecutive days. Twelve collection sites, in immediate proximity to human housing, were chosen. A detailed description of the collection spots has been previously published [20]. *Culex pipiens* complex mosquitoes yielded at collection spot Bbd03 (13.2693939208984, −59.6246032714843) were used for this study. Trapped mosquitoes were shock-frozen at −80°C within a maximum time of 60 min after trap disassembly and transferred to the research laboratory of the University of Veterinary Medicine, Vienna. Female mosquitoes were specified (morphologically) using the key of Becker and (single legs) genetically verified with molecular barcoding, as previously reported [23,24]. None of the individuals included in subsequent analysis showed signs of recent blood meal intake.

### 2.2. Sample Preparation—Virus Purification and Enrichment Protocol (VIPEP) 

Mosquito individuals of each spot were pooled in numbers of 50. Mechanical homogenization was performed using a TissueLyser II (Qiagen, Venlo, The Netherlands) with five 2.8 mm ceramic beads (Peqlab, Erlangen, Germany) and 1 mL of Dulbecco’s phosphate-buffered saline (DPBS) buffer at a frequency of 30 strokes per second for a duration of 4 × 30 s interspersed by a 30 s pause. Virus purification and enrichment protocol (VIPEP) was performed on mosquito homogenates in combination with an additional SpeedVac (Thermo Fisher Scientific, Waltham, MA, USA) concentration step, as previously described [25]. In short, virus particles were enriched by a nine-step procedure that included initial resuspension of the homogenates in DPBS buffer pH7 (Dulbecco’s PBS, no calcium, no magnesium, Thermo Fisher Scientific), two centrifugation steps for 5 min at 2500× *g* and 15 min at 4800× *g*, filtration through a 0.45 μM syringe filter, ultrafiltration using 50 kDa molecular weight cut-off filtration units (Amicon Ultra-15 50K, Merck Millipore, Cork, Ireland), DNase I digestion (Qiagen), DNA and RNA preparation using a QIAamp UCP Micro Kit (Qiagen), blocking of ribosomal RNA sequences using a set of 5 specific oligonucleotides, cDNA synthesis using nonribosomal hexanucleotides together with the Super Script IV enzyme (Thermo Fisher Scientific), and final amplification of the total nucleic acids using a Repli-g kit (Qiagen).

### 2.3. Illumina Library Preparation and Sequencing

MDA-amplified double-stranded cDNA and genomic DNA were quantified with a Qubit dsDNA High Sensitivity Kit on a Qubit 4 fluorometer (Thermo Fisher Scientific) according to the manufacturer’s instructions. Shotgun library preparation was done with a NEBNext Ultra II DNA Library Prep Kit for Illumina in combination with the Index Primers Set 1 and 2 (New England BioLabs, Frankfurt, Germany) according to the manufacturer’s instructions. Briefly, up to 30 ng dsDNA were fragmented by ultrasonication in a Bioruptor Pico sonication system (Diagenode, Liege, Belgium) in a total volume of 55 μL 1× TE for 5 cycles of 15 s on and 30 s off. Then, 50 μL of fragmented DNA was used for end repair and adapter ligation reactions, according to the manufacturer’s instructions, for a DNA input of less than 100 ng. Size selection and purification were performed according to instructions for 500 to 700 bp insert size. Subsequent PCR amplification was performed with 12 cycles, and libraries eluted after amplification and purification in 33 μL 1× TE buffer (pH 8.0). For quality control, libraries were analyzed with a DNA High Sensitivity Kit on a 2100 Bioanalyzer system (Agilent Technologies, Santa Clara, CA, USA) and quantified on a Quantus™ fluorometer (Promega, Walldorf, Germany). Following library preparation, equimolar pools were sequenced on an Illumina MiSeq desktop sequencer (Illumina, San Diego, CA, USA). Libraries were diluted to 8 pM and run without PhiX control for 600 cycles with version three chemistry according to the manufacturer’s instructions. FASTQ Files were used for data analysis.

### 2.4. Bioinformatic Analysis

Raw sequencing data were quality trimmed and adaptors removed using AdapterRemoval v2.2.0 (https://adapterremoval.readthedocs.io), keeping only sequences longer than 30 bp, with a maximum error rate of 3 and trimming ambiguous bases in 3′ and 5′ ends. Trimmed datasets were de novo assembled using metaSPAdes v3.12.0 (https://cab.spbu.ru/spades) with default parameters. Contigs larger than 500 bp were used for downstream analyses. Open reading frames (ORF) were predicted on the contig sequences using MetaGeneMark v3.38. BLASTN analysis (Blast+ v2.5.0) (http://exon.gatech.edu/meta_gmhmmp.cgi) against the NCBI nt database was used as a first approach to determine contig identity, employing a minimum e-value threshold of 1 × 10^−5^ and keeping the first 25 hits. A BLASTP analysis of the predicted ORF sequences (in amino acids) against the NCBI nr database (*e*-value < 1 × 10^−5^, 25 first hits) was used as a second approach. Depending on the case (see the Results section), taxonomic annotation of contigs was resolved by either importing BLAST results to MEGAN v6.10.13, which uses the lowest common ancestor algorithm for classification, or by considering the best BLAST hit. The relative abundance of each contig in each sample was calculated using the RPKM metric (reads per kilobase per million mapped reads), which normalizes by contig lengths and sequencing depth of samples. For this, reads were remapped on contigs using bwa v0.7.10-r789 with the “*aln*” method, while coverage and depth were calculated on the resulting bam files using custom scripts and bedtools v2.26.0 (https://bedtools.readthedocs.io). In addition, the abundance of each individual taxa (based on NCBI taxID) was also estimated using the RPKM method, utilizing the average of RPKM of all contigs assigned to the same taxID.

Vector sequences and hits that were also detected in the negative control were excluded from further analysis. To simplify the taxonomic analysis, best BlastN hits to identical NCBI taxonomy ID were clustered. If more than one contig matched the same best hit, mean pairwise sequence identity (% ID) and mean coverage of query sequence (% subject coverage) were used for the analysis. Alternatively, when contigs were found to hit different sequences but with the same NCBI taxonomy ID, mean % ID and mean % query coverage were calculated for each subject sequence.

### 2.5. Phylogenetic Analysis

Predicted Rep and Cap protein sequences of the novel virus were used for phylogenetic analysis. Amino acid sequences of Rep and Cap proteins of (i) proposed type species of the nine phylogenetic genera of the family of *Genomoviridae*, according to Zhao et al., and (ii) ten other *Genomoviridae* species identified by BlastN were retrieved from the NCBI database [26]. Additionally, the homologous protein sequence of *Gemycircularvirus* type species was included as the outlier. Sequence alignments were created in T-Coffee using the PSI-Coffee protein alignment algorithm, including protein structure information [27]. Alignments of Rep and Cap protein sequences were trimmed to conserve only regions covered by the contig sequence, and trimmed alignments were concatenated for phylogenetic tree construction. 

The phylogenetic tree was generated using the maximum likelihood algorithm based on the LG model including discrete Gamma distribution (+G) and by assuming that a certain fraction of sites are evolutionarily invariable (+I). This model was identified as the best fitting model with the lowest BIC (Bayesian information criterion) in MEGA10 [28,29]. The bootstrap consensus tree was inferred from 100 repetitions. Initial trees for the heuristic search were obtained automatically by applying neighbor-ioin (NJ) and BioNJ algorithms to a matrix of pairwise distances estimated using a JTT model and then selecting the topology with a superior log likelihood value.

### 2.6. Virus-Specific Polymerase Chain Reactions (PCRs)

PCR reactions were performed for verification of metagenomic detection of Bat circovirus POA/2012/II (BatCv) and Cyclovirus VN isolate hcf1 (CyCV-VN). Primers were designed in Primer3 (http://primer3.ut.ee) software, and primer sequences were tested for specificity by Primer Blast analysis (https://www.ncbi.nlm.nih.gov/tools/primer-blast/). Amplicon length was chosen to be less than 200 nt (132 bp for BatCV and 160 bp for CyCV-VN), as required for testing shared sequencing libraries. The following primer pairs were chosen: BatCV_fw (5′- ATCCAGCCGTAGAAGTCGTC-3′) and BatCV_rv (5′-CGGAAAATCAAAGCGTGCAC-3′), CyCV_fw (5′- TGAAGGAGGAGAGACATGCC-3′) and CyCV_rv (5′- TGTTCCAGTCGATCCCCAAA-3′). The PCR mixture contained 1× iTaq PCR buffer (Bio-Rad, Hercules, CA, USA), 200 mM of each deoxynucleotide triphosphate (dNTP) mix, 2 mM MgCl_2_, 5 mM of each forward and reverse primer, and 0.4 U iTaq DNA polymerase (Bio-Rad) in a 20 μL reaction volume. Additionally, 2 μL of VIPEP enriched Ae.ae.BRB or C.pip.cl.BRB was used as a template and 2 μL of nucleic acid-free water as a negative control. PCR reactions included initial denaturation in a thermal cycler at 95 °C for 15 min, followed by 35 cycles of denaturation at 94 °C for 60 s, annealing at 59 °C (BatCV) or 54 °C (CyCV-VN) for 60 s and extension at 72 °C for 2 min, followed by a final extension at 72 °C for 10 min. Amplicons were visualized by electrophoresis on 2% agarose gel.

Verification of human Torquet Teno virus (TTV) was done by real-time PCR (RT-PCR), as previously described by Maggi et al. [30]. Briefly, PCR reactions were done in a total volume of 20 μL that contained 10 μL iTaq Universal Supermix (Bio-Rad), 300 nM concentration of primers (AMTS and AMTAS), 200 nM TaqMan probe (AMTPTU), and 2 μL of template DNA. Mixtures were prepared in 96-well optical microtiter plates (Thermo Fisher Scientific) and amplified on a StepOnePlus real-time PCR system (Thermo Fisher Scientific) by using the following cycling parameters: denaturation for 90 s at 95 °C, followed by 40 cycles of denaturation for 15 s at 95 °C, and annealing and extension for 60 s at 60 °C. A plasmid carrying the amplicon insert was used as positive control, and nucleic acid-free water was used as negative control. All samples were tested in duplicates.

## 3. Results

### 3.1. Highly Sensitive Virome Characterization

**Figure 1:**
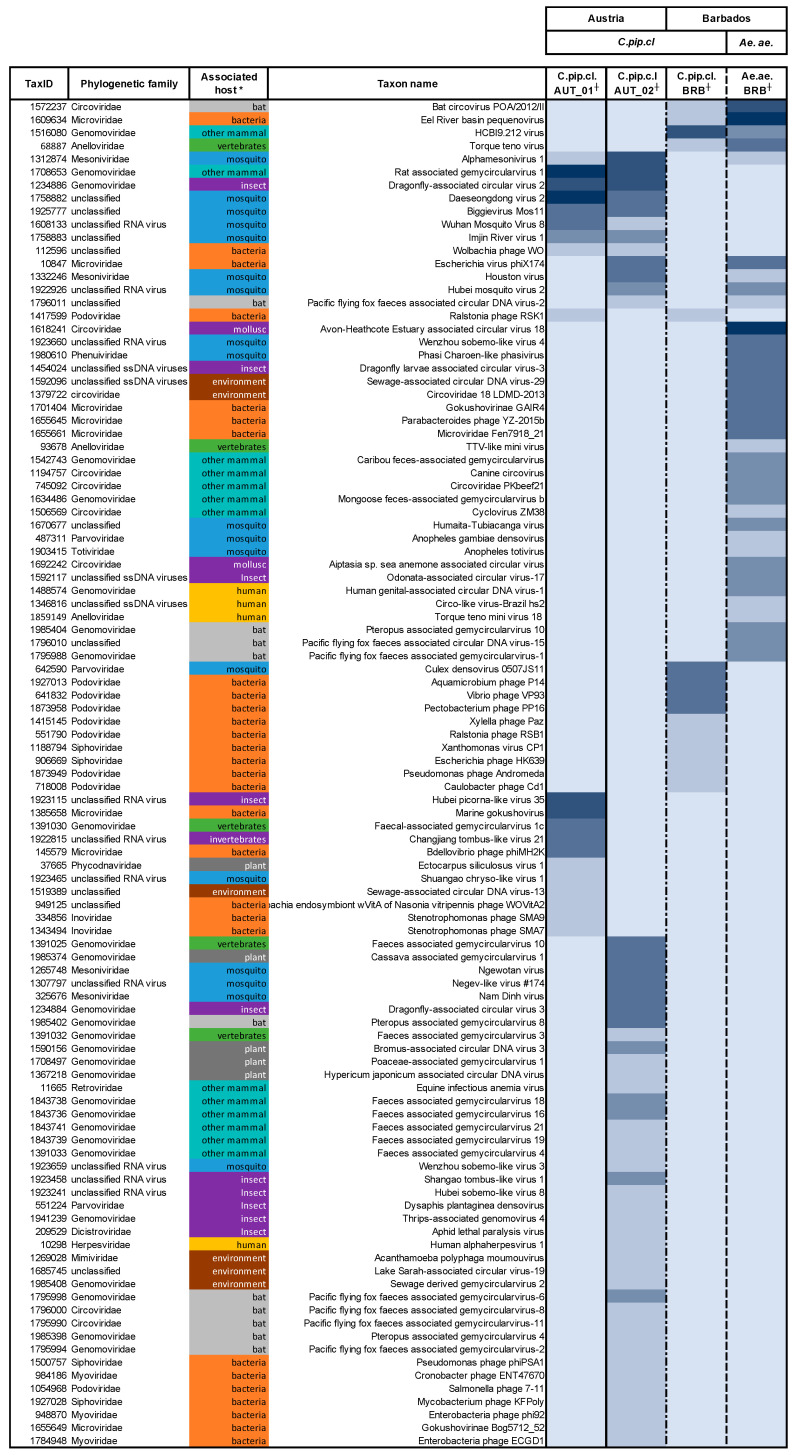
Metagenomic virome richness; * host organisms grouped by color, ^┼^ shades of blue indicate relative abundance in RPKM.

For this study, we used four pools of field-collected mosquitoes from Austria and Barbados (Supplementary Table S1). Highly specific purification of virus nucleic acids was performed for each pool separately, including a reverse transcriptase step to detect DNA and RNA viruses. Virus metagenome sequencing on the Illumina MiSeq platform generated 2.00 × 10^6^ to 1.41 × 10^7^ raw sequencing read pairs per sample; read pairs were consequently trimmed and assembled to contigs. Taxonomic annotation matched 66% to 83% of contigs longer than 500 bp to NCBI database entries (Supplementary Table S1). Efficient presequencing elimination of cells, cell-derived debris, and nonviral sequences yielded highly sensitive sequencing of viral genomes. Hits to plasmid sequences and sequences matching hits that were detected in no-template control samples were removed from the dataset. Eukaryotic and prokaryotic sequences accounted only for a minor fraction (3–12%) of total assigned sequences, and the majority of reads could be assigned to viral genomes (88–97%) (Supplementary Figure S1). Viral contigs that were assigned to broad categories, such as uncultured or unclassified viruses, were excluded from further analysis. We found that mosquitoes hosted a highly diverse set of viruses that differed greatly between the pools. The overall richness was high, with 103 different viral taxa identified at the species level, of which 86 were detected in a single sample (Figure 1). We further assessed virome richness at the family taxonomic level. We found that the mosquito virome in all four pools was predominantly constituted by families from the class of circular CRESS-DNA viruses (Supplementary Figure S2), which were also the most abundant fraction in all pools (Supplementary Figure S3).

### 3.2. Influence of the Environment on the Mosquito Virome

Mosquito pools from similar environments were found to share mutual virus hits, referred to as ecosystem signature viruses. *C.pip.cl.* mosquito pools from Austria (AUT_01 and AUT_02) shared eight viral hits while mosquitoes from Barbados (Ae.ae.BRB and C.pip.cl.BRB) shared a set of four mutual viral hits (Figure 1). We did not find any evidence that mosquitoes of the same species complex from Austria and Barbados carried common viral infections. Moreover, the virome of *Cx. pipiens* complex mosquitoes from Barbados was more similar to *Ae.aegypti* mosquitoes from the same ecosystem (four mutual viral taxa) than compared to the pools of *Cx. pipiens* complex mosquitoes from Austria (one mutual viral taxon with C.pip.cl.AUT_01). However, these findings are limited by the small number of pools tested.

We then analyzed the impact of the immediate surrounding environment within the range of the average flight distance of *Cx. pipiens* complex mosquitoes from the collection spots. Governmental land coverage maps were used to characterize the immediate surroundings (Supplementary Figure S4) [20]. Although the number of sites was relatively limited, we did not observe a higher virome richness in more diverse environments. For example, the area surrounding collection spot AUT_01 was considerably more diverse (comprising seven different categories of land coverage) and included a higher proportion of natural-state areas (58.1%) such as waterbodies, urban green lands, and recreational areas than found at spot AUT_02 (37.1%) (Supplementary Table S2). However, the relative abundance of viral reads (97%) and the overall richness of viral taxa (58) was higher in pool AUT_02 than in pool AUT_01 (Supplementary Figure S1).

### 3.3. The Mosquito Virome is Comprised of Viruses from A Wide Diversity of Hosts

**Figure 2:**
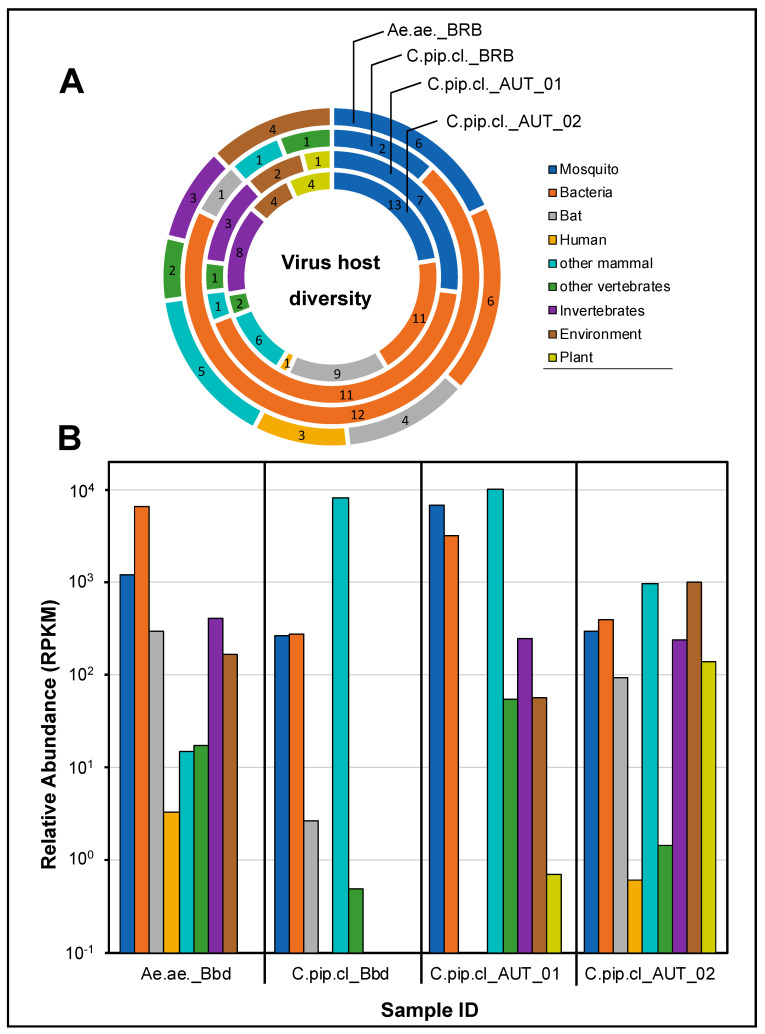
All obtained hits to viral taxa grouped by the associated host organism (NCBI). (**A**) Richness in number of hits to indicated host group for each mosquito pool; (**B**) accumulative relative abundance (RPKM) of viral hits grouped by host organism.

To further characterize the mosquito virome, we analyzed virus hits for their associated host species annotated in the NCBI database. We identified viruses across a wide range of hosts in both *Ae. aegypti* and *Cx. pipiens* complex mosquitoes. Besides viruses of vertebrate hosts, such as human, bat, and other mammal viruses, we detected viruses associated with invertebrate hosts, plant viruses, environmental viruses, and bacteriophages. When viral taxa were clustered according to related host groups, we observed that taxonomic richness was highest for mosquito-associated viruses and bacteriophages (Figure 2A). Within the group of vertebrate hosts, viruses associated with mammals and, in particular, to bats prevailed at high diversity. These two groups were among the most abundant throughout all four samples (Figure 2B). However, this finding might be biased by the fact that the viral microbiome of bats has been sampled more extensively than that of other wild mammal species.

### 3.4. Mosquito-Specific Viruses form the Core Group of the Mosquito Virome

We separated metagenomic sequence assignments to their goodness-of-assignment fit by plotting pairwise sequence identity versus query coverage in a two-dimensional scatterplot. Viral hits to broader categories were included in this analysis to gain further information on sequence similarity, regardless of classification. To test this method of analysis, we used a 100-nucleotide model sequence from an NCBI database virus genome sequence (LK931484.1) and introduced variable numbers of random point mutations between 10% to 50% of total length (Supplementary Table S3). Assignment of sequences with mutation rates greater than 30% resulted in hits, which were taxonomically unrelated to the primary sequence (e.g., plants, fish, bacteria). Sequences with 25–30% of mutated nucleotides were at the borderline of being correctly annotated (Supplementary Table S4). Assignments to taxonomically unrelated taxa were based on BLAST alignments with high pairwise sequence identity, though covering only a minor fraction of query sequence. When plotted, biologically implausible hits clustered clearly apart from correct assignments, separated by a query coverage of less than 60%. Hits to taxonomically related taxa (i.e., virus sequences) fell within close proximity to the group of correct assignments but with lower pairwise sequence identity (Supplementary Figure S5). 

**Figure 3:**
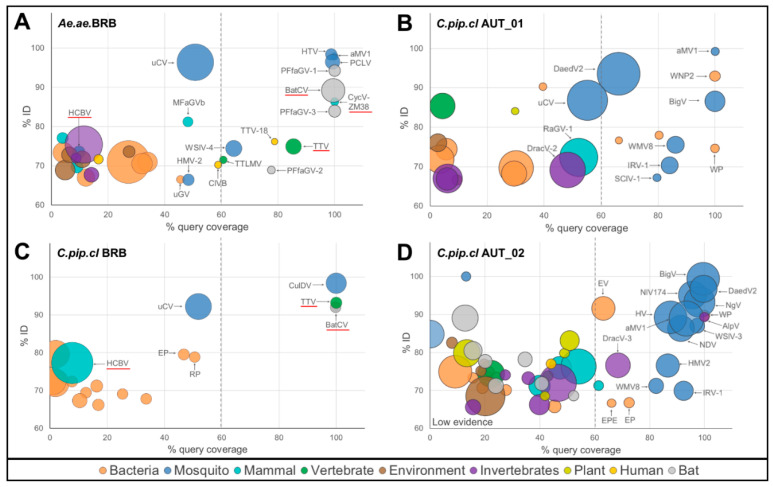
Goodness-of-assignment fit for viral metagenomes of (**A**) *Aedes aegypti* mosquitoes, Barbados, (**B**) *Culex pipiens* complex mosquitoes, Austria pool 01, (**C**) *Culex pipiens* complex mosquitoes, Barbados, (**D**) *Culex pipiens* complex mosquitoes, Austria pool 02. Bubbles represent metagenomic viral hits separated by % coverage of query sequence (*x*-axis) and % pairwise sequence identity (*y*-axis), size of bubbles represents relative abundance (RPKM), color indicates associated virus host; underlined sequences have been verified by PCR assay. aMV, alphamesoniviurs 1; AlpV, aphid lethal paralysis virus; BatCV, bat circovirus POA/2012/II; BigV, biggievirus Mos11; ClVB, circo-like virus, Brazil hs2; CulDV, *Culex* densovirus 0507JS11; CycV-ZM38, cyclovirus ZM38; DaedV2, Daeseongdong virus 2; DracV-2, dragonfly-associated circular virus 2; DracV-3, dragonfly-associated circular virus 3; EP, Escherichia phage HK639; EV, Escherichia virus phiX174; HMV-2, Hubei mosquito virus 2; HCBV, HCBI9.212 virus; HTV, Humaita–Tubiacanga virus; HV, Houston virus; IRV-1, Imjin River virus 1; MFaGVb, mongoose feces-associated gemycircularvirus b; NDV, Nam Dinh virus; NgV, Ngewotan virus; NlV174, Negev-like virus #174; PCLV, Phasi Charoen-like virus; PFfaGV-1, Pacific flying fox feces-associated gemycircularvirus-1; PFfaGV-2, Pacific flying fox feces-associated gemycircularvirus-2; PFfaGV-3, Pacific flying fox feces-associated gemycircularvirus-3; RaGV-1, rat-associated gemycircularvirus 1; RP, Ralstonia phage RSK1; SClV-1, Shuangao chryso-like virus 1; TTV, Torque teno virus; TTV-18, Torque teno virus 18; TTLMV, TTV- like mini virus; uCV, uncultured circovirus; uGV, uncultured Gokushovirinae; WP, Wolbachia phage WO; WN2P, Wolbachia endosymbiont wVitA of Nasonia vitripennis phage 2; WMV8, Wuhan mosquito virus 8; WSlV-3, Wenzhou sobemo-like virus 3; WSlV-4, Wenzhou sobemo-like virus 4.

We used this method of analysis to identify those hits of the mosquito virome that closely resembled assigned virus taxa (Figure 3). Among this group of high-likelihood hits (>60% query coverage) mosquito-associated viruses were the predominant virus group besides hits to bacteriophages, bat-associated viruses, and a few vertebrate viruses. Of six high-likelihood hits to bacteriophages, two resembled *Wolbachia*-infecting phage species. Moreover, we found that ecosystem signature viruses were predominantly assigned with a high degree of confidence. 

For the two Austrian mosquito pools, six out of eight ecosystem signature viruses were assigned with query coverage >60 (Figure 3B,D). This group of viruses was mainly formed by mosquito-specific viruses (5 out of 6) and two *Wolbachia*-infecting bacteriophages. Daesongdong virus 2 (DaedV2) was previously identified in *C.pip.cl*. mosquitoes from South Korea, while different strains of Biggievirus Mos11 (BigV) have been identified in *C.pip.cl*. mosquitoes from the US, Italy, and India (GI KX924639, MF281708, MF281709, MH603566). *Imjin River virus 1* (*IRV1*), an ssRNA virus taxonomically related to *Wuhan mosquito virus 8* (WMV8), has been previously identified in virus metagenomes of *Cx. bitaeniorhynchus* from South Korea [31]. For mosquitoes from Barbados, we identified two mutual viruses that lay within the range of >60% query coverage (vertebrate-infecting *Torquet Teno virus* (*TTV*) and *Bat circovirus POA/2012/II*). Notably, the fraction of CRESS-DNA viruses in the group of high-confidence hits, across all samples, was less than in the overall metagenomes (34.9% vs. 55.2%; Supplementary Table S5).

### 3.5. Validation of Metagenomic Results

We validated metagenomic sequencing results by using molecular detection methods. To verify single surrogate CRESS-DNA hits with a high goodness-of-assignment fit from the Ae.ae.BRB virome (Bat circovirus POA/2012/II, Cyclovirus ZM38, and Torquet Teno virus), we designed specific PCR assays on the respective metagenomic sequences. All four PCR assays yielded uniformly positive results, and sequencing of PCR amplicons verified NGS-derived metagenomic sequences (Supplementary Figure S6).

CycV-ZM38 resembles a strain of *Cyclovirus VN* (*CyCV-VN*) virus species. Sequence analysis of the contig sequence annotated as CycV-ZM38 revealed a pairwise sequence identity to CyCV-VN of 78%, including a 141 nt sequence that is identical to the last 141 nt (1855–1995) of the contig, indicative of the circular genomic structure. The contig sequence displayed a conserved genome organization, with two ORFs resembling a capsid protein (cap) coding gene and a replicase (Rep) coding gene in BlastP analysis. According to the species demarcation criteria for circoviruses, being <75% of genome nucleotide identity, the metagenomic sequence resembles a closely related, novel variant of CyCV-VN.

### 3.6. Novel Viruses Were Mostly CRESS-DNA Viruses 

Hits with 0–60% query coverage matched database sequences only at short stretches, thereby resembling widely novel sequences. Of these, 64.9% were assigned as CRESS-DNA viruses (Supplementary Table S5). Compared to the total dataset, hits with less than 60% of query coverage were significantly enriched in CRESS-DNA viruses (chi-square test, R = 10.6, *p* = 0.001).

High-fidelity virus assignments were associated with hosts that fit into the biological context (i.e., mosquitoes, bats, symbiotic bacteria). Hits of lower goodness-of-assignment fit corresponded to hosts that were inappropriate to the ecosystem of mosquito collection (e.g., caribou feces-associated gemycircularvirus). Moreover, environmental viruses, plant viruses, and the majority of bacteriophage sequences were clustered in this field of low goodness-of-assignment fit. 

**Figure 4:**
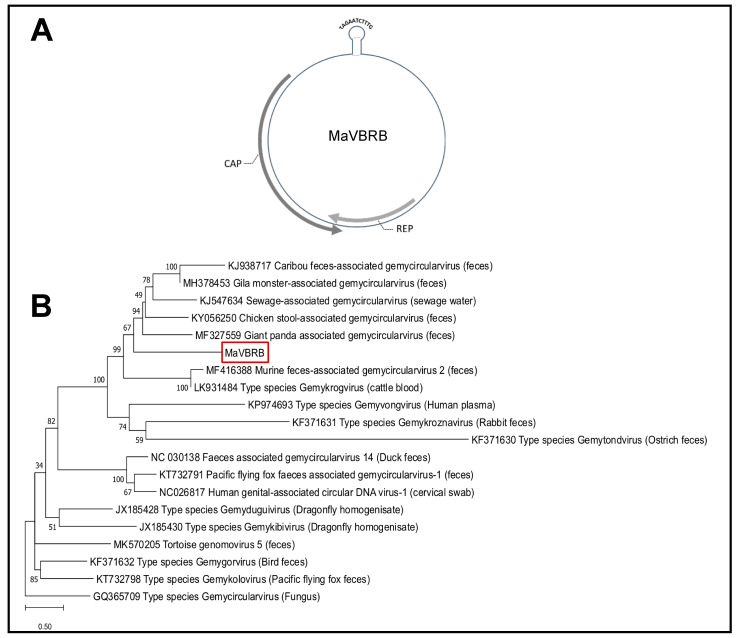
(**A**) Genome organization of novel *mosquito-associated virus Barbados* (*MaVBRB*) consensus sequence identified in Ae.ae.BRB and C.pip.cl.BRB mosquito pools. (**B**) Phylogenetic tree of members of the family of *Genomoviridae* using replication-associated protein (Rep) and capsid protein (Cap) sequences, tree branches indicate sequence GI and primary source of sequence isolation; *MaVBRB* clusters in the genus of *Gemykrogvirus* type species.

As seen for the model sequence in Supplementary Figure S5, mutation rates exceeding 30% in a random database sequence mislead BLASTN sequence annotation to taxonomically unrelated taxa. However, this model-simulated accumulation of random point mutations assumed a completely nonconserved genome sequence. This cannot be assumed for viral genomes that include protein-coding regions or other conserved genome segments. Hits with low goodness-of-assignment fit are particularly susceptible to a change in their annotation alongside the expansion of databases, as seen for the HCBI9.212 virus. At the time of metagenome analysis for this study (10/2018), two contig sequences from the Ae.ae.BRB and C.pip.clBRB metagenomes were assigned as the HCBI9.212 virus (query coverage 10% and 7.8% and pairwise similarity 73% and 77%, respectively). Since then, the NCBI database has been constantly expanded by novel annotations, assigning both contigs, at the time of writing (02/2020), as apis mellifera genomovirus 2, with increased goodness-of-assignment fit (Supplementary Figure S7). However, further investigation of the contig sequence revealed that the short aligning sequence was located at the capsid protein gene sequence, highly conserved in the family of *Genomoviridae.* The full-length contig sequence *k141_1014* (2222 nucleotide) resembled the circular genome of a novel CRESS-DNA virus of the family of *Genomoviridae*, tentatively named *mosquito-associated virus Barbados* (*MaVBRB*; Figure 4A). Comparing the *k141_1014* sequence to the C.pip.cl.BRB-derived metagenomic sequence assigned as HCBI9.212, we found that the underlying contig sequence shared 99.5% pairwise sequence identity to the MaVBRB genomic sequence from the *Ae.ae.* pool. The sequence included two inversely oriented open reading frames coding for a replication-associated protein (Rep) and a capsid protein (Cap) and presented the structural features of two palindromic sequences flanking a putative origin of replication. In the phylogenetic tree constructed using Rep amino acid sequences from viruses of the family of *Genomoviridae*, MaVBRB clusters were within the genus of *Gemykrogvirus* (Figure 4B). Covering the total sequence with 5 abutting-primer PCRs, we confirmed the NGS-derived genomic virus sequence (Supplementary Figure S8).

## 4. Discussion

Mosquitoes are the most important vectors for the global transmission of arthropod-borne diseases that account for more than 700,000 deaths annually [32]. Studying the viral microbiome of medically important mosquito vectors can help us to better understand virus dynamics and epidemiology of known and novel human pathogenic viruses. 

In this study, we analyzed and compared virome patterns of two medically relevant mosquito species (*Cx. pipiens* complex. and *Ae. aegypti)* trapped at distinct ecosystems (Austria and Barbados). We assembled a large diversity of viral sequences matching database entries of viruses that were previously associated with a range of hosts. Separating hits by their goodness-of-assignment fit, we provide evidence that mosquito vectors host a “core virome” that is closely related to previously identified virus taxa. Strikingly, a large fraction of this set of viruses was shared among mosquito pools from the same ecosystem, even though collection points were separated by several kilometers in distance. Ecosystem signature viruses of the Austrian pools predominantly resembled close taxonomic relatives of mosquito-specific viruses that have been identified previously in *Culex* mosquitoes from different parts of the world [31,33]. Hence, our data support the previous host association of DaedV2 and BigV to *C.pip.cl.* and emphasize the global abundance of these mosquito-specific viruses. However, despite the limiting number of samples, we observed more similarity in viromes from closely related environments than in the same mosquito species from different environments. We, therefore, hypothesize that the local ecosystem may play an important role (arguably, more important than the host species) in shaping the viral communities in mosquitoes as individuals of different species are exposed to common sources of virus infection.

Moreover, we found evidence that different *Wolbachia*-infecting bacteriophage strains are part of the viral microbiome of sampled Austrian *C.pip.cl*. mosquitoes. *Wolbachia* are intracellular alphaproteobacteria, often living in endosymbiosis with arthropods. Infection with endosymbiotic *Wolbachia* has been shown to alter the reproductive behavior of its arthropod host [34]. Therefore, Wolbachia-infecting prophage WO has been recently proposed as a beneficial tool for vector control efforts [35]. This is the first report of *Wolbachia-infecting prophage WO* (Tax ID 112596) and *Wolbachia phage WOVitA2* (Tax ID 949125) in field-collected mosquitoes from central Europe. However, we did not recover full genomic sequences as corresponding contigs covered just parts of the bacteriophage genomes that might represent conserved sections of this taxonomic group. 

Analyzing the viral metagenome on a taxonomic level, we found that CRESS-DNA viruses account for a disproportionally prominent fraction of the taxonomic richness and form the most abundant taxonomic group in the mosquito virome. The taxonomic clade of Rep-encoding ssDNA viruses (CRESS-DNA viruses) is highly diverse and abundant. CRESS-DNA viruses share a similar genome organization, all encoding a highly conserved replication-associated protein (Rep) and at least one further, less conserved capsid (Cap) protein. In many virus metagenomic studies, multiple displacement amplification (MDA) is used as a standard method for unspecific sequence amplification, whereby small circular ssDNA sequences such as CRESS-DNA viral genomes are preferentially amplified. Hence, it has to be taken into account that relative virus representation might thereby be biased [36]. However, the usage of MDA has uncovered the vast abundance of CRESS-DNA viruses prevailing almost ubiquitously throughout most ecosystems, including the human body.

Among CRESS-DNA viruses of vertebrate hosts, bat viruses accounted for a disproportionally high fraction of the *Ae. ae.* virome. Bat circovirus POA/2012/II (BatCv POA/II) is a CRESS-DNA virus of the family of *Circoviridae* that was initially detected in bat feces collected in Southern Brazil [37]. Besides this first metagenomic identification of BatCv POA/II, there have been no other detections to differentiate whether the virus effectively replicates in bats or if the virus simply passes through the digestive tract of these insectivorous animals, as previously suggested [37]. Hence, identification of BatCV POA/II in two mosquito pools of different species indicates that the virus might primarily infect mosquitoes rather than bats. Likewise, this observation was discussed for other families of CRESS-DNA viruses, as two out of three phylogenetic clusters of *Cycloviruses* may only infect arthropods while they are currently being associated with diverse vertebrate hosts [38]. All four bat-associated viruses of the *Ae. ae.* virome were exclusively identified by metagenomic sequencing of bat feces. Final host assignment can only be done by further virus isolation experiments. However, the current findings suggest that a spillover of CRESS-DNA viruses does not only occur from vertebrate hosts downwards onto mosquitoes but also from mosquitoes upwards. Thereby, mosquitoes act as mixing vessels for CRESS-DNA viruses from different environmental sources, which may promote recombination events leading to novel virus variants.

CRESS-DNA viruses evolve rapidly, with comparable evolution rates to RNA viruses [39]. High mutation rates and the possibility for genome recombination are consistent with a high speed of CRESS-DNA virus evolution [18,26,40,41]. By bringing together viruses from different hosts, mosquito vectors might provide ideal preconditions for recombination events of highly abundant CRESS-DNA viruses. By mapping hits by their pairwise sequence identity and query coverage, we were able to identify metagenomic sequences that most likely match known viruses from sequences that represent novel virus sequences. We provide evidence that the group of novel viruses in the mosquito virome is predominantly formed by unknown CRESS-DNA viruses. Of those, we picked one hit to characterize the viral genome of a novel *Gemykrogvirus*, tentatively named MaVBRB. MaVBRB forms a phylogenetic cluster with a later identified AmGV-2, infecting honeybees. Most of the other members of the genus of *Gemykrogviruses* have previously been detected in the feces of various animal species [39]. Since virus–host specificity is commonly conserved among viral families, the identification of MaVBRB and AmGV-2 might provide an important lead for prospective host assignment of *Gemykrogviruses*. It is still a matter of speculation what the true hosts of *Gemykrogviruses* are. One possibility is that *Gemykrogviruses* actually infect plants, which would explain their detection in the feces of herbivorous animals and in mosquitoes that feed on pollen. 

The host range of eukaryotic CRESS-DNA viruses is highly diverse and includes plants and various invertebrate and vertebrate species, including humans [26,42]. Indeed, human Torquet Teno virus (TTV), a CRESS-DNA virus from the *Anelloviridae* family, accounts for the most abundant virus infection of humans, with estimated worldwide infection rates of 70–100% [43,44,45]. In this study, we identified TTV genomic sequences in two separate mosquito pools collected in Barbados and verified the metagenomic finding by qPCR (specific for human TTV species). Finding human-infecting TTV in field-caught mosquito populations suggests a spillover of CRESS-DNA viruses to mosquitoes, most likely transmitted by feeding on the blood of infected vertebrate hosts. It may be assumed that the detected mosquito virome diversity is driven by viruses of different sources that passage through the individual mosquito’s digestive tract. CO2-baited trapping selects for unfed mosquitoes, and none of the included mosquito individuals showed morphologic signs of recent blood meal intake. However, it cannot be totally excluded that CRESS-DNA virus detection might have been influenced by the remainder of vertebrate blood within the individuals.

Moreover, single CRESS-DNA viruses have been identified in serum and brain biopsies of multiple sclerosis patients, in the pericardial fluid of pericarditis patients, and the cerebrospinal fluid of encephalitis patients [46,47,48]. CyCV-VN was initially identified in cerebrospinal fluid (CSF) from patients with suspected central nervous system infection from Vietnam, as well as in feces from humans and pigs from the same area [48]. The pathogenic role of CyCV-VN remains unclear as screening of >600 CSF patient samples from Vietnam, Cambodia, Nepal, and the Netherlands failed to detect the virus [49]. However, in later studies, CyCV-VN was detected in stool samples of healthy children from Madagascar, pig feces from Cameroon, and shrew enteric samples from Zambia [50,51]. A recent study reported a 43% plasma prevalence of CyCV-VN in healthy blood donors from Madagascar [52]. In this study, we identified a novel genetic variant of CyCV-VN. To our knowledge, this is the first study to detect CyCV-VN in mosquito vectors, possibly suggesting transmission via *Aedes aegypti*. However, further studies are needed to confirm these findings in larger cohorts and to investigate mosquito vector competence for CyCV-VN transmission. 

In conclusion, this study comprehensively characterized the viral metagenome of field-collected *C.pip.cl.* and *Ae. ae.* mosquitoes from two distinctive geographic locations. Among all phylogenetic virus clades, CRESS-DNA viruses constituted a diverse and highly abundant portion of the mosquito virome. We present evidence that mosquito vectors are important hubs for CRESS-DNA virus transmission between different environmental sources. Thus, mosquitoes contain a large pool of novel CRESS-DNA viruses from which we identified a novel variant of a human-infecting CRESS-DNA virus, currently not linked to vector-related transmission.

## Supplementary Materials

The following are available online at https://www.mdpi.com/2076-0817/9/9/686/s1, Figure S1: Fraction of reads mapping on contigs classified as viruses, prokaryotes and eukaryotes, Figure S2: Virome composition on taxonomic family level as number of hits to indicated clade, Figure S3: Relative abundance of viral hits on taxonomic family level in RPKM, Figure S4: Land coverage of Austrian collection spots AUT_1 and AUT_2, Figure S5: Goodness of alignment- fit of best BLAST N hits for for mutated model sequences, Figure S6: Verification of metagenomic hits, Figure S7: Goodness of assignment- fit for best BLAST N hit of contig sequence k141_1014 from *Aedes aegypti* (*Ae.ae.*) metagenome and contig sequence NODE_38 from *Culex pipiens* complex (*C.pip.cl*.) Barbados (BRB) using NCBI database version 2018 and updated database version 2020; Figure S8: Verification of metagenomic assembled *mosquito associated virus Barbados* (*MaVBRB*) genome sequences by abutting primer PCR reactions; Table S1: Sequencing results, sequence separation and quality trimming, Table S2: Land coverage, AUT_01 and AUT_02, Table S3: Model sequences of 100 nucleotide length from primary NCBI entry LK931484.1 with indicated number of random point mutations, Table S4: Best BLAST N hits of model sequence to NCBI database, Table S5: Number of CRESS-DNA viruses hits below and above 60% query coverage.
