# Supplementary material for: Highly Sensitive Virome Characterization of Aedes aegypti and Culex pipiens Complex from Central Europe and the Caribbean Reveals Potential for Interspecies Viral Transmission
Source: Pathogens. 2020 Aug 21;9(9):686. doi: 10.3390/pathogens9090686 (PMC7559857; doi:10.3390/pathogens9090686)
Supplement: Supplementary file 1 [file pathogens-09-00686-s001.zip › 2020-08-21 Supplementary files/Figure S1-S8.pdf]

Figure S1

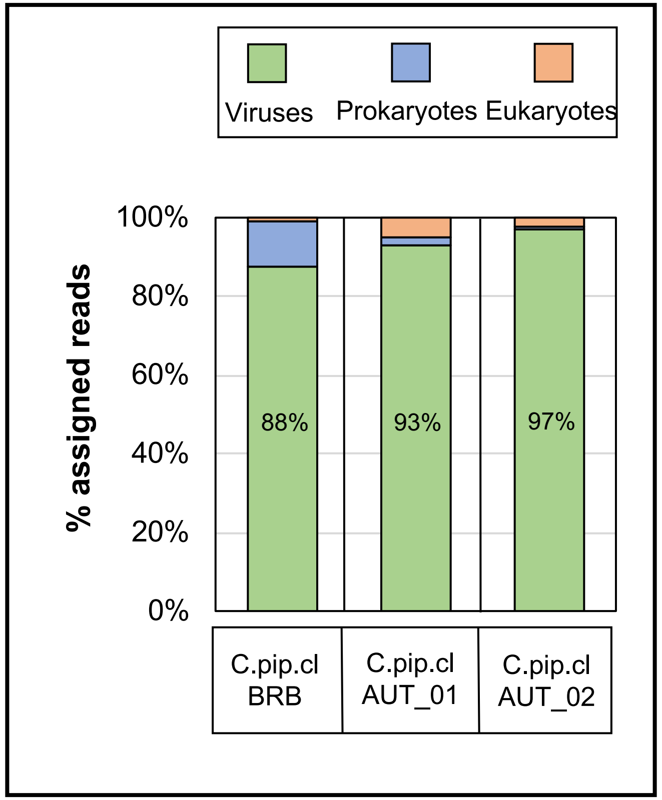

Figure S1 Fraction of reads mapping on contigs classified as viruses, prokaryotes and eukaryotes.

Figure S2

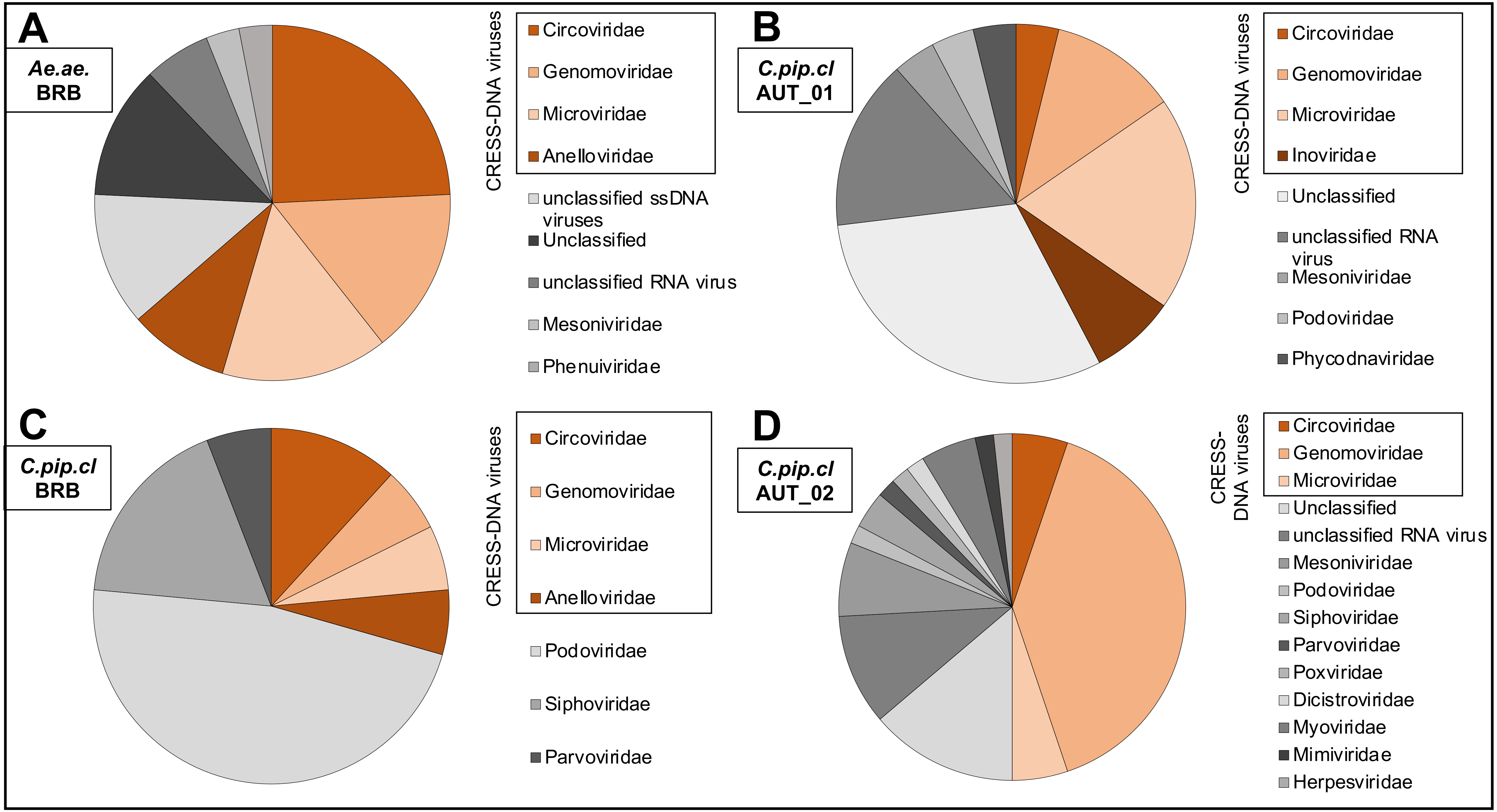

Figure S2 Virome composition on taxonomic family level as number of hits to indicated clade; families of the group of circular rep encoding single stranded (CRESS)- DNA viruses are marked in shades of red;

Figure S3

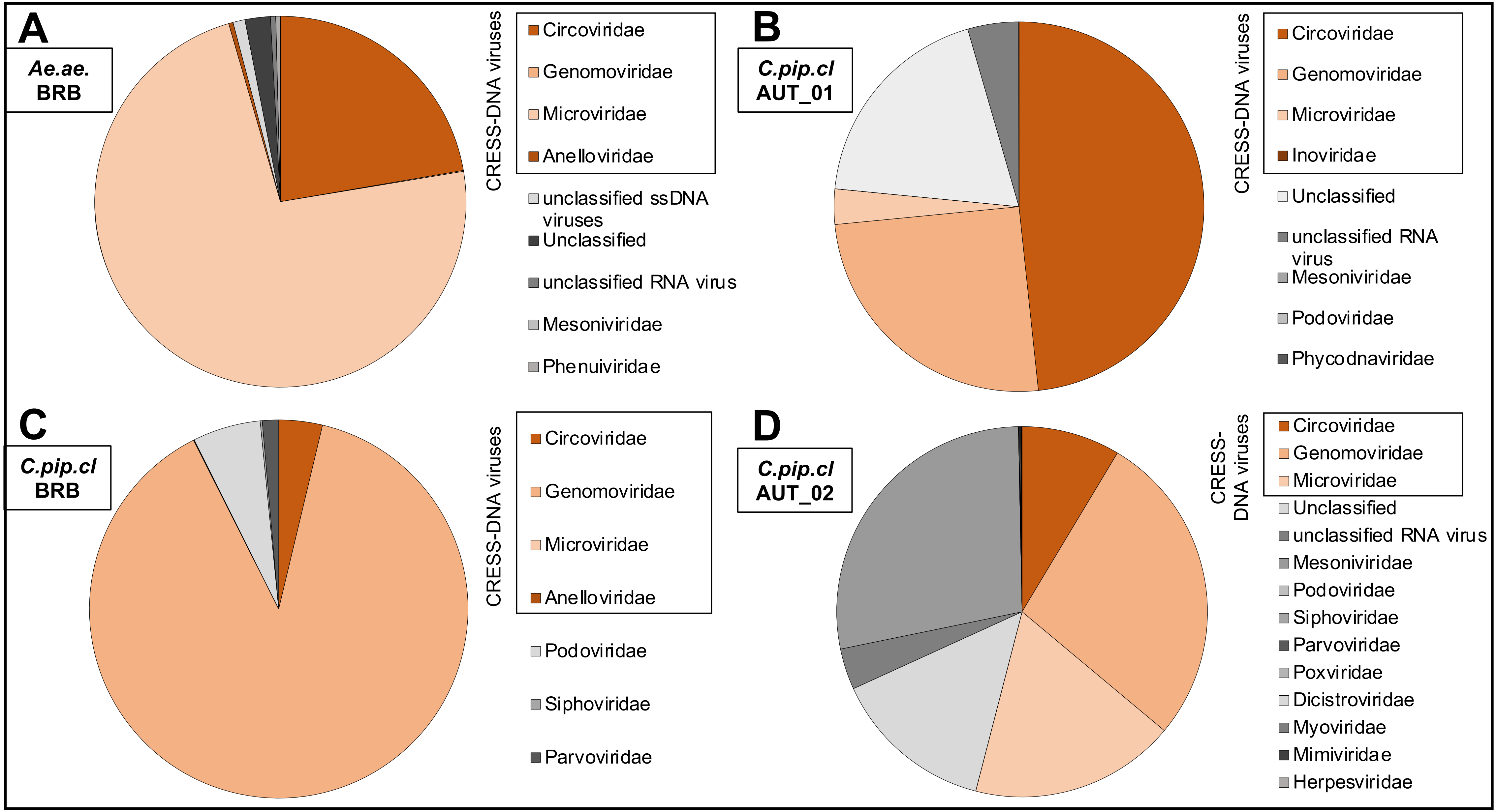

Figure S3 Relative abundance of viral hits on taxonomic family level in RPKM; families of the group of circular rep encoding single stranded (CRESS)- DNA viruses are marked in shades of red;

Figure S4

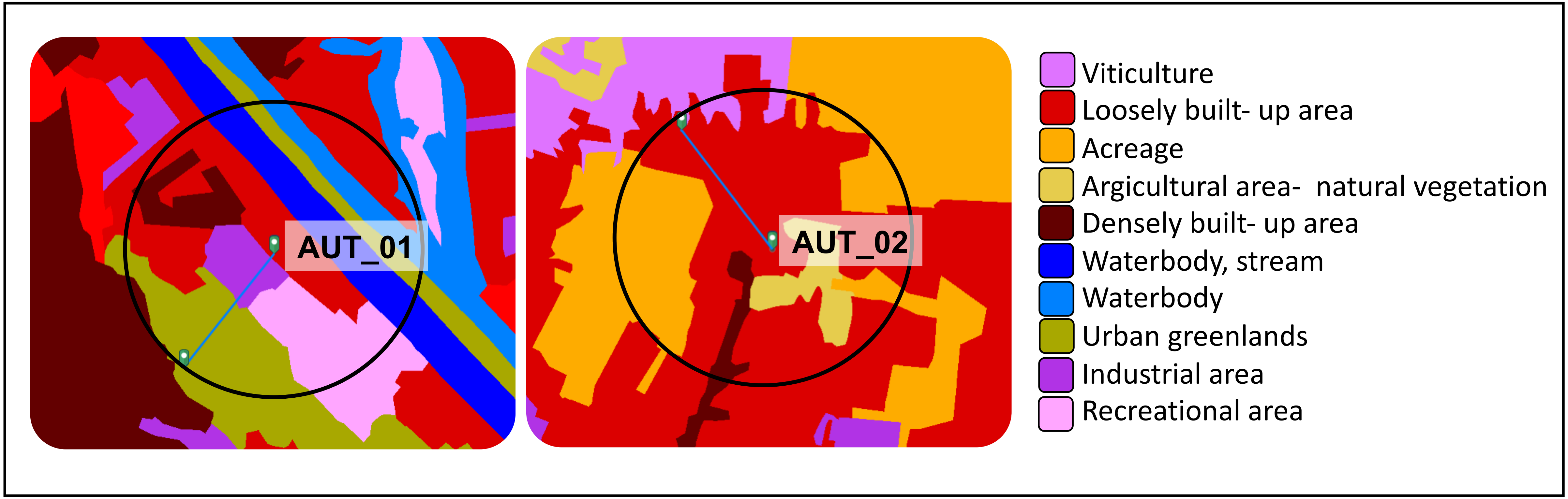

Figure S4 Land coverage of Austrian collection spots AUT_1 and AUT_2; circle represents area covered by mean flight distance of 1.3 km from point of collection of *C.pipiens* complex mosquitoes (approx 1.33 km)

Figure S5

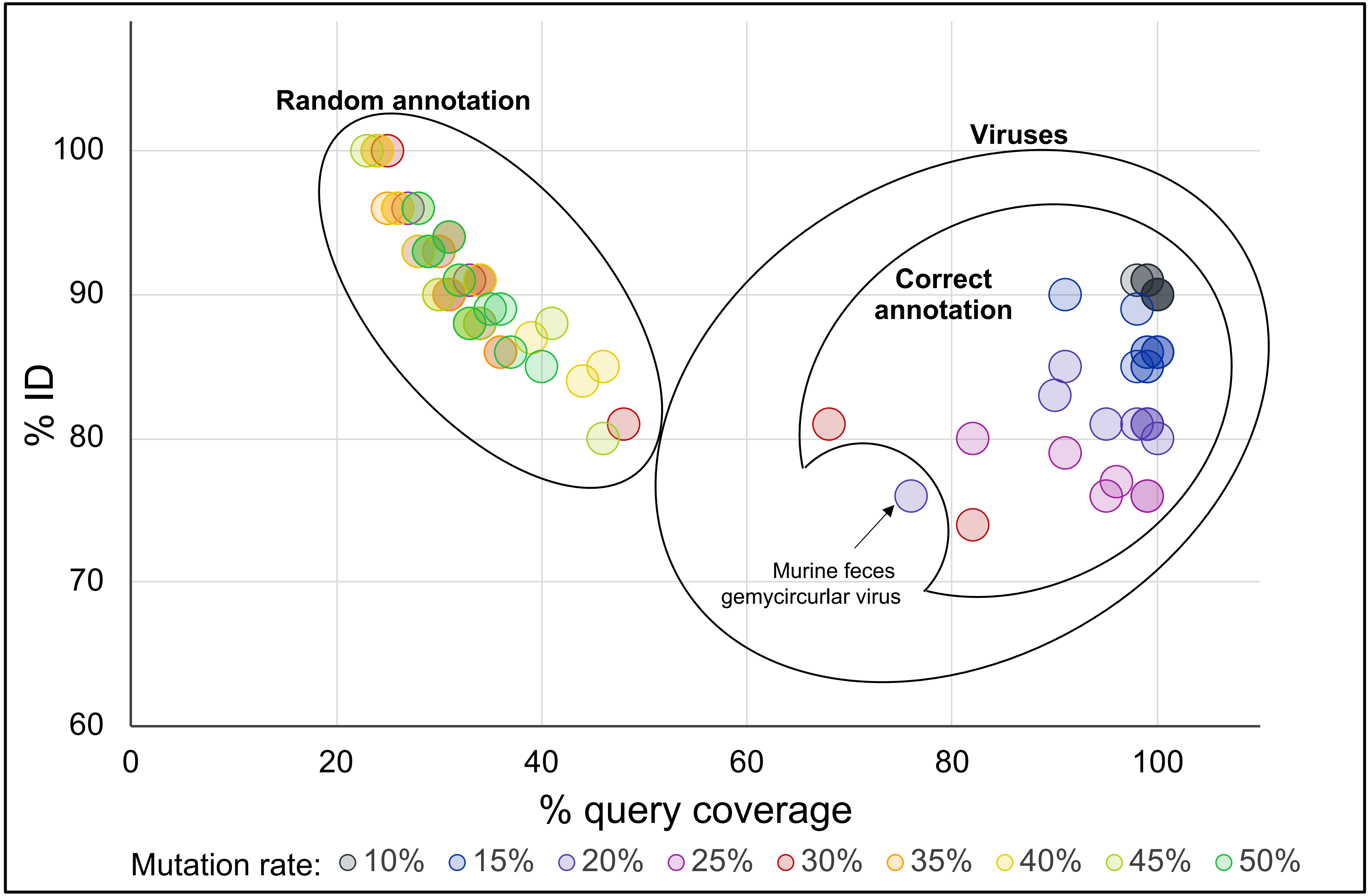

Figure S5 Goodness of alignment- fit of best BLAST N hits for for mutated model sequences

Figure S6

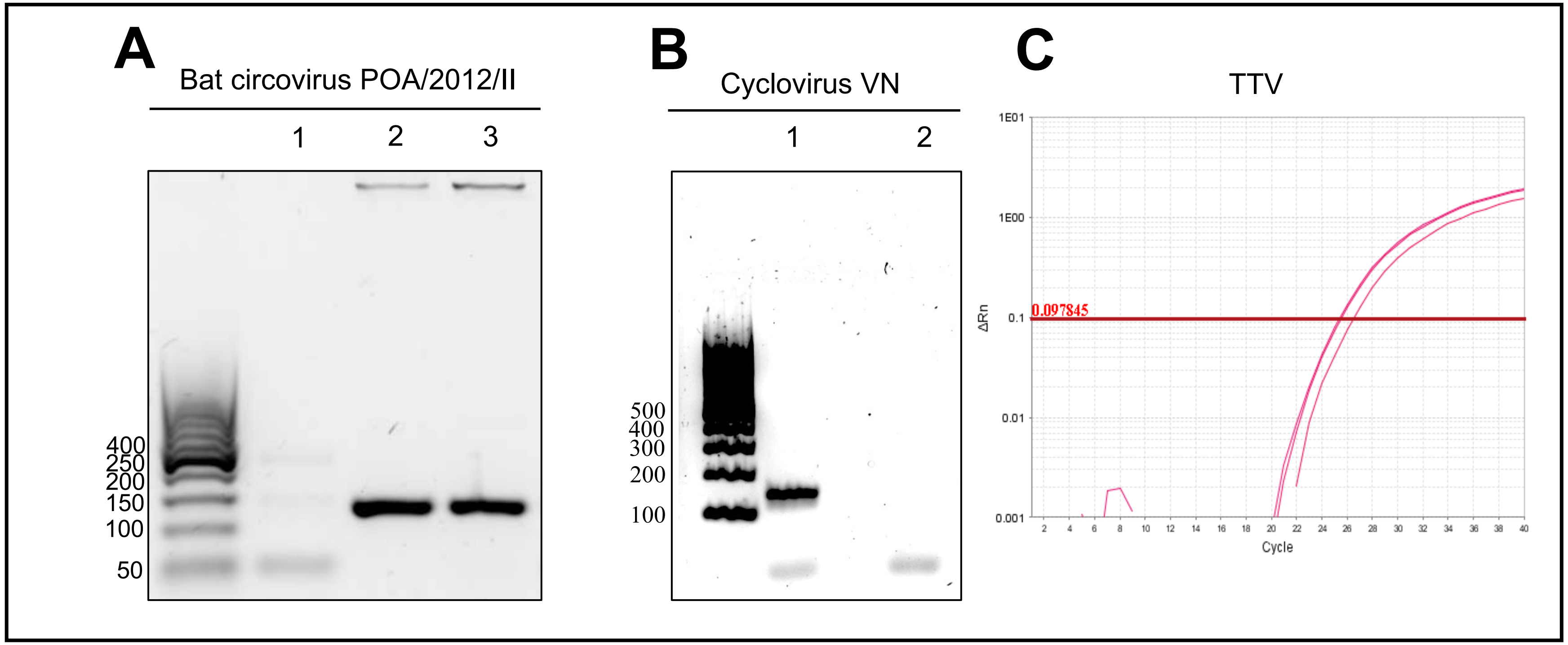

Figure S6 Verification of metagenomic hits by (**A**) specific PCR assay designed to target a 132bp sequence (pos 965-1096) of Bat circovirus POA/2012/II (NC_025791); lane 1: negative control; lane 2: *Ae.ae.* BRB; lane 3: *C.pip.cl.* BRB; (**B**) specific PCR assay designed to target a 160bp sequence (pos 574-714) of Cyclovirus VN isolate hcf1 (NC_021707); lane 1: *Ae.ae.* BRB; lane 2: negative control; (**C**) specific taqMan rtPCR assay designed to target a conserved 63 bp sequence among human infecting TTV genome sequences

Figure S7

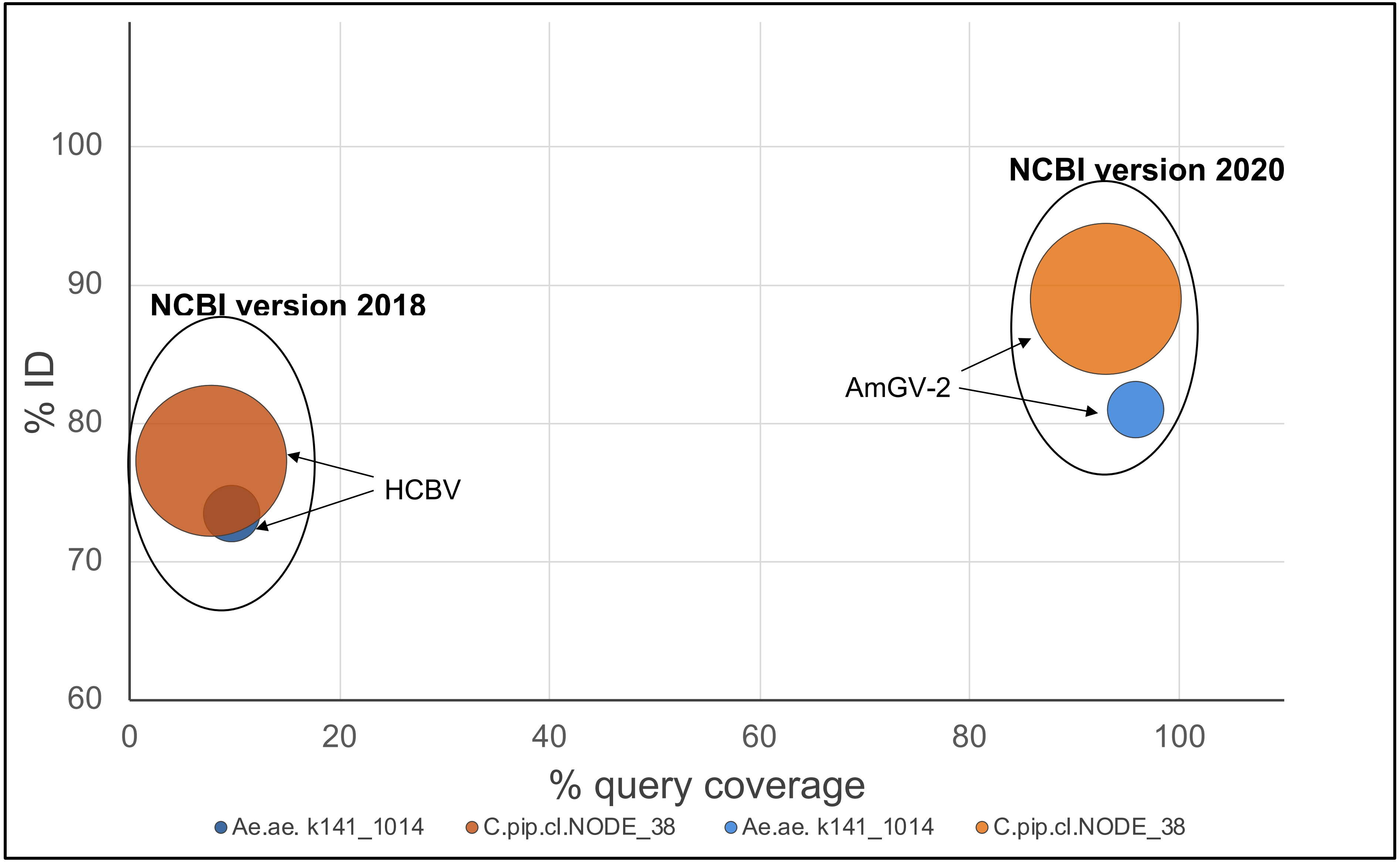

Figure S7 Goodness of assignment- fit for best BLAST N hit of contig sequence k141_1014 from *Aedes aegypti* (*Ae.ae.*) metagenome and contig sequence NODE_38 from *Culex pipiens* complex (*C.pip.cl.*) Barbados (BRB) using NCBI database version 2018 and updated database version 2020;

Figure S8

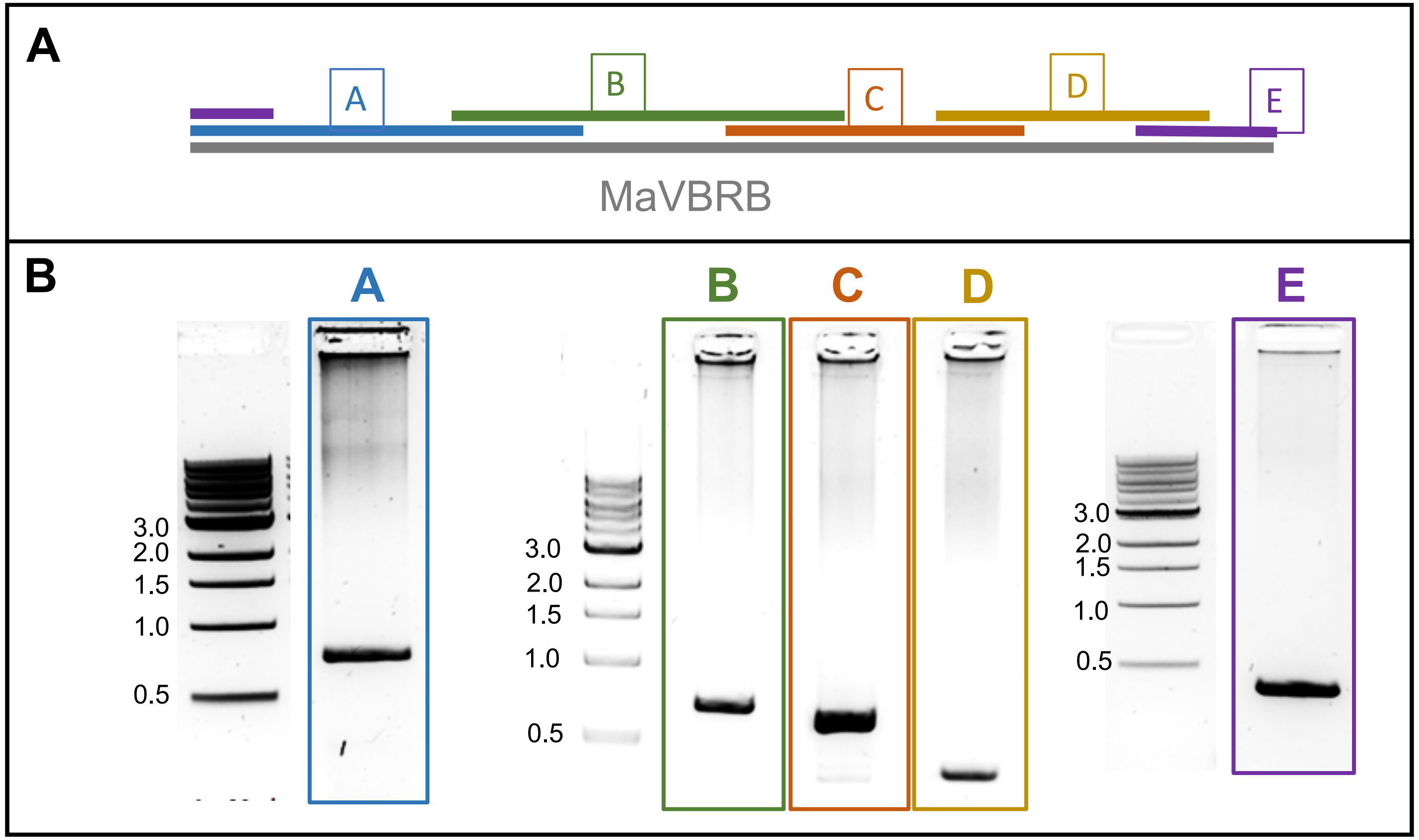

Figure S8 Verification of metagenomic assembled *mosquito associated virus Barbados* (*MaVBRB*) genome sequences by abutting primer PCR reactions; (**A**) position of PCRs on genomic virus sequence and (**B**) gel separation of PCR products
