# Supplementary material for: Highly Sensitive Virome Characterization of Aedes aegypti and Culex pipiens Complex from Central Europe and the Caribbean Reveals Potential for Interspecies Viral Transmission
Source: Pathogens. 2020 Aug 21;9(9):686. doi: 10.3390/pathogens9090686 (PMC7559857; doi:10.3390/pathogens9090686)
Supplement: Supplementary file 1 [file pathogens-09-00686-s001.zip › 2020-08-21 Supplementary files/Table_S1.pdf]

Table S1; Sequencing results, sequence separation and quality trimming

ID	Mosquito species (pool size)	Country of origin	Latitude	Longitude	Decription	date	raw read pairs	trimmed reads	assembled reads (% of trimmed)	average bp/contig	BlastN contigs >500bp	BlastN contigs matched database entries (% of trimmed)
C.pip.cl_AUT_1	*Culex pipiens* complex (50)	Austria	48.215766	16.413106	casern, semi rural	04.08.16	3.66E+06	5.16E+06	4.02E+06 (78)	1,207	828	648 (78)
C.pip.cl_AUT_2		Austria	48.29397	16.419941	cortyard, urban	17.08.16	1.41E+07	2.14E+07	4.14E+06 (19)	621	6.564	4688 (71)
C.pip.cl_BRB		Barbados	13.269393	-59.624603	private garden, rural	31.10.16	2.00E+06	2.69E+06	2.33E+06 (87)	1,090	812	677 (83)
Ae.ae_BRB	*Aedes egypti* (27)	Barbados	as reported previously in Thannesberger et.al. 2019 (18)				2.06E+06	3.34E+06	2.43E+06 (73)	2.214	162	107 (66)
