# Supplementary material for: Highly Sensitive Virome Characterization of Aedes aegypti and Culex pipiens Complex from Central Europe and the Caribbean Reveals Potential for Interspecies Viral Transmission
Source: Pathogens. 2020 Aug 21;9(9):686. doi: 10.3390/pathogens9090686 (PMC7559857; doi:10.3390/pathogens9090686)
Supplement: Supplementary file 1 [file pathogens-09-00686-s001.zip › 2020-08-21 Supplementary files/Table_S2.pdf]

Table S2; Land coverage, AUT_01 and AUT_02

AUT_01				
		absolute area	relative area (%)	summ (%)
natural state areas	Waterbody- stream	0.567	14.3	
	Waterbody	0.279	7.1	
	Urban greenlands	0.917	23.2	
	Recreational area	0.534	13.5	*58.1*
built- up state areas	Loosely built- up area	1.141	28.9	
	Densely built- up area	0.285	7.2	
	Industrial area	0.231	5.8	*41.9*
	total	*3.954*	*100.0*	

AUT_02				
		absolute area	relative area (%)	summ (%)
natural state areas	Viticulture	0.065	1.6	
	Acreage	1.066	26.9	
	Agriculture- natural vegetation	0.339	8.6	*37.1*
built- up stat e	Loosely built- up area	2.372	59.9	
	Densely built- up area	0.116	2.9	*62.9*
	total	*3.958*	*100.0*	
