# Supplementary material for: Highly Sensitive Virome Characterization of Aedes aegypti and Culex pipiens Complex from Central Europe and the Caribbean Reveals Potential for Interspecies Viral Transmission
Source: Pathogens. 2020 Aug 21;9(9):686. doi: 10.3390/pathogens9090686 (PMC7559857; doi:10.3390/pathogens9090686)
Supplement: Supplementary file 1 [file pathogens-09-00686-s001.zip › 2020-08-21 Supplementary files/Table_S3.pdf]

Table S3 Model sequences of 100 nucleotide length from primary NCBI entry LK931484.1 with indicated number of random point mutations

**sequence 1**	**LK931484.1; 1-100**	ATTTTTCGACCAGTAAAAAATGGCTTACGGATTCCGCCGCCGCTTTGGTCGCTCCCGTCGCTCGTACCGACGAAGGCCCGTTCGCCGCTATTCCCGAAGG
Seq1.1	10 Mut	ATTTATCGACCACTATAAAATGCCTTCCGGATACCGCCGCCGCTTTGGTCGCTCCCGTCGCTCGTACCGACTAAGGCCCGTTCGCCGCTATTACTGACGG
	15 Mut	CTTTATCGACCACTATAAAATGCCTTCCGGATACCGCCGCTGCTTTGCTCGCTCCCGTCGCTCGTACTGACTAAGGCCCGTTCGCCGCTATTACTGCCGG
	20 Mut	CTTTATCGACCACTATAAAATGCCTTCCGGTTACCGACGTTGCTTTGCTCGCTCCCGTCGCTCGTACTGACTAATGCCCGTTCGCCGCTATTAATGCCGG
	25 Mut	CTTTATCGACCACTATAAAATGCCTTCCGGTTACCAACGTTGCTCTGCTCGCTCCAGTCGCTCGTACTGACTAATGCCCATTCGCCGCAATTAATGCCGG
	30 Mut	CTTTATCGAAAACTATAAAATGCCTTCCGGTTACCAACGTTGCTCTGCTCGCTCCAGACGCACGTACTGACTAATGCCCATTCGCCGCAATTAATGCCAG
	35 Mut	CTTTATAGAAAACTATAACATGCCTTCCGGTTACCAAAGTTGCTCTGCTCGCTCCAGATGCACGTACTGACTAATGCCCATTCGCCTCAATTAATGCCAG
	40 Mut	CTTTATAGTAAACTATAACATGCCTTCCGGTTACCAAAGTTGCTCTTCTCGCTCCAGATGCATGTTCTGACTAATGCCCATTCGCCTAAATTAATGCCAG
	45 Mut	CTATATAGTAAACTATAACATGCCTTCCGGTTACCAAAGTTGCTCTTCTCGCCCCAGATGCATGTTTTGAATTATGCCCATTCGCCTAAATTAATGCCAG
	50 Mut	CTATATAGTAATCTATAACATGCCTCCCGGTTACCAAAGTTTCTCTTCCCGCCCCAGATGCATGTTTTGAATTATGCCCATTCGCCTAAATTAATACCAG
Seq1.2	10 Mut	ATTTATCGACTTGTAAAAAATGGCTTACGTATTCCGCCGCCGCTATGGTCGCTCCCGCCGCTCGTACCGTCGAAGGACCGTTCGCAGCTATTCCCAAAGG
	15 Mut	ATTTATCGACTTGTATAAAATGACTTACGTATTCCGCCGCCGCTATGGTTGCTCCCGCCGCTCGTACAGTCGAAGGACCGTTCGCAGCTATTCCCAAACG
	20 Mut	ATTTATCGACTTGTATAAAATGACTTACGTATTCCGCTGCCGCTATGGTTGCTCCCGCCGCTCATACAGTCGAAGGATCGTTCGCAGCTCTTCCAAAACG
	25 Mut	ATTTATCGACTTGTATCAAATGACTCACGTATTCTGCTGCCGCTATGGTTGCTCCTGCCGCTCATACAGTCGAAGGATCGTTCGCAGCTCTTCCAAATCG
	30 Mut	ATTTATCGACTTGAATCAAATGACTCACTTATTCTGCTGCCGCTATGGTTGCACCTGCCGCTCATACAATCGAAGGATCGTTCGCAGCTCTTTCAAATCG
	35 Mut	ATTTATCGACTTGAATCAAATGATTCACTTATTCTGATGACGCTAAGGTTGCACCTGCCGCTCATACAATCGAAGGATCGTTCGCAGCTCTTTTAAATCG
	40 Mut	ATTTATCGCCTTGAATCAAATGATTCTTTTATTCTGATGACGCTAAGGTTGCACCTGCCGCTCATATAATCGAAGGATCGTTCACAGCTCTTTTAAATCG
	45 Mut	ATATAACGCCTTGAATCAAATGATTCTTTTATTCTGATGACGCTAAGGTTGCACCTGCCGCTAATATAATCGAAGGATCATTCACAGATCTTTTAAATCG
	50 Mut	ATATAACGCCTTGAATCAAATGATTCTTTTATTATTATGACGCTAAGGTTGCACCTGCAGCTAATCTAATCGAATGATCATTCACAGATCTTTTAAATCG
Seq1.3	10 Mut	CTTTTTCGACCAGCAATAAATGGCTTACTGATACCGCAGCCGCTTTGGTCGCTCCCGTCGCTCGTACCCACGAAGGCTCGTTCGCCGCTCTTCCTGAAGG
	15 Mut	CTTTTTCGACAAGCAATAAATGGATTACTGATACCGCAGCCGCTTTGGTCGCTCCCGTCGCTCGTACCCACGCAGGCTCGTTCGTCGCTCTTCCTGACGG
	20 Mut	CTTTTTCGACAAGCCATAAATTGATTACTGATACCGCAGCCGCTTTGGTCGCTTCCGTCGCTCGTAACCACGCAGGCTCGTTCATCGCTCTTCCTGACGG
	25 Mut	CTTTTTCGACAAGCCATAAATTGATTACTGATAACGCAGCTGCTTTGGTTGCTTCCGTCGCTCGTAACCACGCAGGCTCGTTCATCGTTCTTCCTGACCG
	30 Mut	CTTCTTCGACAAGCCATAAATTGATTACTGAAAACGCAGCTGCTTTGGTTGCTTTCGTCGCTCGTAACCACTCAGGCTCGTTCATCGTACTTCCTGACCG
	35 Mut	CTTCTACGACAAGCCATAACTTGATTAATGAAAACGCAGCTGATTTGGTTGCTTTCGTCGCTCGTAACCACTCAGTCTCGTTCATCGTACTTCCTGACCG
	40 Mut	CTTCTAACACAAGCCATAACTTGATTAATGAAAACCCAGTTGATTTGGTTGCTTTCGTCGTTCGTAACCACTCAGTCTCGTTCATCGTACTTCCTGACCG
	45 Mut	CTTCTAACACAAGCCATTACTTGATTAATGAAAACCCAGTTGATTCGGTTGCCTTCGCCGTTCGTAACCACTCAGTCTCGTTCATAGTACTTCCTGACCG
	50 Mut	CTTCTAACACAAGCCTTTACTTGATTAATCAAAACCCAGTTGATTCGGTTGCCTTCTCCGTTCGTAACCACTCAGTCTTGATCATAGTACTTCCTGACCG
Seq1.4	10 Mut	ATTTATCGACCAGTAACAAATGGCTCACGGATTACGCCGCCGCTTTGGTCGCTCCCGCTGCTCTTACTGACGAAGGCCCTTTCGCCGCTATTCCCGCAGG
	15 Mut	ATTTATCCACCAGCAACAAATGGTTCACGGATTACGCCGCCGCTTTGGTCGCTCCCGCTGCTCTTACTGAAGAAGGCCCTTTCGCTGCTATTCCCGCAGG
	20 Mut	ATATATCCACCTGCAACAAATGGTTCCCGGATTACGCCGCCGCTTTGGTTGCTCCCTCTGCTCTTACTGAAGAAGGCCCTTTCGCTGCTATTCCCGCAGG
	25 Mut	ATATATCCACCTGCAACAAATGGTTCCCGGATTACACCGCCGCTTTGGTTGCTCCCTCTGCTCTTTATGAAGAAGGCCCTTTCGCTTCTATTACCGCAGG
	30 Mut	ATATATCCACCTGCAACAAATGGTTCCCGGATTACACCGCCGCTTTGGTTGCTCACTCTGATCTTTATGAAGACGGCCCTTTCGATTCTATTACCGCCGG
	35 Mut	ATATATCCACCTGCAACAAATGGTTCCCGGATTACACCGCAGCTTTGTTTGCTCATTCTGATCTTTATGAAGACGGCCCTCTCGATTCTATTACCACCGG
	40 Mut	ATATATCCACCTGCAACAAATGGTTCCCTGATTACACCGCAGCCTTGTTTCCTCATTCTGATCTTTATGAAGACGGCCCTCTCGATTCTATAACCACCTG
	45 Mut	ATATATCCACCTGCAACAAACATTTCCCTGTTTACACCGCAGCCTTGTTTCCTCATTCTGATCTTTATGAAGACGGCCCTCTCGATTCTATAACCACCTG
	50 Mut	TTATATCCACCTGCAACAAACATTTCCCTGTTTACACCGCAGCCCTGTTTCCTCATTCTGATCTATATGTAGACGGCCATCTCGATTCCATAACCACCTG
Seq1.5	10 Mut	AATTTTCGTCCCGTAACAAATTGCTTACGGATTTCGCCGCCGCTTTGGTCGCACCCGTCGCTAGTACTGCCGAAGGCCCGTTCGCCGCTATTCCCGAAGG
	15 Mut	AATTTTCGTCCCGTACCAAATTGCTTACGGATTTCGCCGCCTCTTTGGTCGCACCCGTCGTTAGTACTGCCGAAGGCCAGTTCGCCGATATTCCCGAAGG
	20 Mut	AATTTTCGTTCCGTACCAAATTGTTTACGGATTTCGCTGCCTCTTTGGTCGCACCCGTCGTTAGTACTGCCGAAGGCCAGATCACCGATATTCCCGAAGG
	25 Mut	AATCTTCGTTCCGTACCAAATTGTTTACGGATTTTGCTGCCTCTTTGGTAGCACCCGTCGTTAGTACTACCGAAGGCCAGATCACCGATATTCCCGACGG
	30 Mut	AACCTTCGTTCCGTACCAAATTGTTTCCGGATCTTGCTGCCTCTTCGGTAGCACCCGTCGTTAGTATTACCGAAGGCCAGATCACCGATATTCCCGACGG
	35 Mut	AACCTTCGTTCCGTACCAATTTGTTTCCGGATCTTGCTGCCTCTTCGGTAGCACCTGTCGTAAGTATTACCGAAGGTCAGAACACCGATATTCCCGACGG
	40 Mut	TACCTTTGTTCCGTACCAATTTGTTTCCGGATCTTGCTGCCTCTTCGGTAGCACATGTCGTAAGTATTACCGAAGGTCAGAACACCTATATTCCTGACGG
	45 Mut	TACCTTTGTTCCTAACCAATTTGTATCCGCATCTTGCTGCCTCTTCGGTAGCACATGTCGTAAGTTTTACCGAAGGTCAGAACACCTATATTCCTGACGG
	50 Mut	TACCATTGTTCCTAACCAATTTGTATCCGCATCTTGATGCCTCTTCGGTAGCACATGTTGTAAGTTTTACCGAAAGTCAGAACACCTATATTCCTGACGG
**sequence 2**	**LK931484.1; 10921-11021**	TAGTAATACTCCTTCTATTGCTAGATTTGCTCCGACTGGGACTCGTTACTGGACGGAGGGTTAGATACAATACAAATTGGATCCACCACATTGATTATGT
seq2.1	10 Mut	TAGTAATACCCCTTCTATTGCTAGACTTGCTCCGACTGGGACTCGTTACTGCACGGACGGCTAGATACAACAAAAATTAGATCCACACCATTGATTATGT
	15 Mut	TAGTAATATCCCTTCTATTGCTAGACTTGCTCCGACTGTGACTCATTACTGCACGGACGGCTAGATACAACAAATATTAGACCCACACCATTGATTATGT
	20 Mut	TAGTAATATCCCTTCTATTGTTAGCCTTGCTCTGACTGTGACTCATTACTGCACGGACGGCTAGATACAACAAATACTAGACCCACACCATTGTTTATGT
	25 Mut	TAGTAATATCACTTCTACTGTTTGCCTTGATCTGACTGTGACTTATTACTGCACGGACGGCTAGATACAACAAATACTAGACCCACACCATTGTTTATGT
	30 Mut	TAATAATTTCACTTCTACTGTTTGCCTTGATCTGATTGTGACTTATTACTGCACGGACGGCTAGATACAACAAATACTAGACCCACACCATTGTCTATCT
	35 Mut	TCATAATTTCACTTCTACTGTTTGCCTTGATCTGATTGTGACTTATTACTGCACGGACGGCTCGATACATCAAATACTAGACCCTCACCATTGTCTAACT
	40 Mut	TCATAATTTCACTTCTACTGTTTGCCTTGATCTGATAGTGACTTATTACTGCACGGTCTGCTCGATACATCAAATACTAGACCCTAACCATCGTCTAACT
	45 Mut	TCATAATTTCACTTCTACTGTTTGCCTTAATCTGATAGTGACTTATTACTGCATAGTCTGCTCGATACATCCAATACTAGACTCTAACCATCGTCTAACT
	50 Mut	TCATCATTTCACTTCCACTGTTTGCCTTAATCTGATAGTGACTTATTATTGCATAGTCTGCTCGATACATCCAATACAAGACTCTAACTATCGTCTAACT
seq2.2	10 Mut	CAGTAATACTCCATCTATTGCTAGATCTGCTCCGACTGGGACTCGTTACTGCACAGAGGGTTTGATATTATACCAATTGGATCCACCACATTCATTATGT
	15 Mut	CAGTAATACTCCATCTATTGCTAGATCTGCTCCGACTGGGACTCATTCCTGCCCAGAGGGTTTGATATTATACCCATTGGATCAACCACATTCATTATGT
	20 Mut	CAGTAATACTCCATCTATCTCTAGATCTGCTCCGACTGGGACTCATTCCTGCCCAGAGGGTTTGATATTATACCCTTTGAATCAACCACATTCCTTATGT
	25 Mut	CAGTAATACTACATCTATCTCTAGATCTGCTTCGACTGGGACTCATTCCTGCCCAGAGGGTTTGATATTCTACCCTTTGAATAAACCACTTTCCTTATGT
	30 Mut	CTGTAATACTACATCTATCTCTAGATCTGCTTCGACTGGGATTCATTCCTGCCCAGAGGGTTTGATATTCTACCCTTTGAAAAAACCACTTACCTTATCT
	35 Mut	CTGTAATACTACACCCATCTCTAGATCTGCTTCGACTGGGTTTCATTCCTGCCCAGAAGGTTTGATATTCAACCCTTTGAAAAAACCACTTACCTTATCT
	40 Mut	CTGCAATACTACACCCATCTCTAGATCTGCATTGACTTGGTTTCATTCCTGCCCAGAAGGTTTGCTATTCAACCCTTTGAAAAAACCACTTACCTTATCT
	45 Mut	CTGCACTACTATACCCATCTCTAGATCTGCATTGACTTGGTTTCATTCCTGCCCAGAAGGTTTGCTATTCAACCCTTATATAAAACCACTTACCTTATCT
	50 Mut	CTGCACTACTATACCCATCTCTAGATCTGCATTGAATTGGTTCCATTCCTGCCCAGAAGGTTTGCTCTTCACTCCTTATATAAAACCACTTACCTTATCT
seq2.3	10 Mut	TATTAATACTCCTTCTATTGCTAGATTTGCTCCGACTGGGACTCGTTTCTGGACGAAGGTTACAATACAATACAAATTGGATCTATCACATTGATTTTGT
	15 Mut	TATTAATACTCCTTCTATCGCTAGATTTGCTCCGAATGGGCCTCGTTTCTTGACGAAGGTTACAATACAATCCAAATTGGATCTATCACATTGATTTTGT
	20 Mut	TATTAATACTCCTTCTATCGCAAGATTTGCTCCGAATGGGCTTCGTTTATTGACGAAGGTTACAATACAATCCAACTTGGTTCTATCACATTGATTTTGT
	25 Mut	TATTAATACCCCTCCTATCGTAAGATCAGCTCCGAATGGGCTTCGTTTATTGACGAAGGTTACAATACAATCCAACTTGGTTCTATCACATTGATTTTGT
	30 Mut	TATTAATACCCCTCCTATCGTACGATCAGCACAGAATGGGCTTCCTTTATTGACGAAGGTTACAATACAATCCAACTTGGTTCTATCATATTGATTTTGT
	35 Mut	TATTAATACCCCTCCTATCGTACGATCAGTACAGAATGGGCTACCTTTATTGACGAAGGTTACAACACATTCCAACTTGGTTCTATCATATTCATTTTGT
	40 Mut	TATTTATACCCCTCCTATCGTACGATCAGTACAGAATGGGCTATCTTTATTGACGAAGGTTACAACACATTCCAACTAGTTTCTATAATATTCATTTTGT
	45 Mut	TATTTTTACCCCTCCTATCGTACGATCAGTATAGAATGGGCTATCTTTATTGACGAATGTTACAACACATTCCAACTACTTTCTATAATATTCATTTTGT
	50 Mut	TATTTTTCCCCCTCCCATCGTACGATCAGTATAGAATGGGCTATCTTTATTGACGAATGTTACAACACTTTCCAACTACTTTATATAATATTCACTTTCT
seq2.4	10 Mut	AAGTAATACTCCTTATATTGCTAGTTTTGCTCCGACAGGGACTCGCTACTGGACAGAGGGTCAGAAACAATTCAAATTGGATCCACCACATTGACTATGT
	15 Mut	AAGTAATACTCCTTATATTGCTAGTAATGCTCCGACAGGGAATCTCTACTCGACAGAGGGTCAGAAACAATTCAAATTGGATCCACCACATTGACTATGT
	20 Mut	AAGTAATACTCCTTATATTGCTAGTAATGCTCCGACAGGGAACCTCTACTCGACACAGGGTCAGAAATAATTCAAATTGGAACCACCACATTTACTATGT
	25 Mut	AAGTAATACTCCTTATATCGTTATTAATGTTCCGACAGGGAACCTCTACTCGACACAGGGTCAGAACTAATTCAAATTGGAACCACCACATTTACTATGT
	30 Mut	AAGTAATACTCTTTATATCGTTATTAATGTTCCGACAGGGAACCTCTACTCGACACAGGGTCTGAACTAATTCAAATTGGAACCACTACTCTTACTATGT
	35 Mut	AAGTAAAACTCTTTATATCGTTATTAATGTTCCGACAGGGAACCTCTTCACGACACAGGCTCTGAACTAATTCAAATCGGAACCACTACTCTTACTATGT
	40 Mut	AAGTAAAACTCTTTATATCGTTATTAATGTTTCGATAGGGAACCTCTTCACGATACAGGCTCTGAACTCATTCAAATCGGAACCACTCCTCTTACTATGT
	45 Mut	AAGTAAAACTATTAATATCGTTCTTAATGTTTCGATAGGGAACCTCTTCACGATACAGGCTCTGAACTCATTAAAATCGGAACCACTCCTCTTACTATGA
	50 Mut	AAGCAAAACTATTAATATCATTCTTAATGTTTCGATAGGCCACCTCTTCACGATACAGGCTCTGAACTCATTAAAATCGGAACCACTCCTCTTTCTATGA
seq2.5	10 Mut	TAGAAATACTCCTTATACTACTAGATTTGCTCCGACTGGGATTCGTTACTGGACGGAGGGTTAGATACAATACTAACTGAATCCTCCACATTAATTATGT
	15 Mut	TAGAAATTCTCCTTATACTACTAGATTTGCTCCGTCTGGGATTCGTTACTGGACGGAGGGTTAGATACAAAACTAACTGAATCCTCCACATCAATTATGC
	20 Mut	TAGAAATTCTCCTTATACTAATAGATTTGCTCCGTCTGGGATTCGTTACTGGACCGAGGGTTAGATACAAAACTAACTGAATCCTTCACAACAATTCTGC
	25 Mut	AAGAACTTCTCCTCATACTAATAGCTTTGCTCCGTCTGGGATTCGTTACTGGACCGAGGGTAAGATACAAAACTAACTGAATCCTTCACAACAATTCTGC
	30 Mut	AAGAACTTCTCCTCATACTAAAAGCTTTGCTCCCTCTGGGATTCGTTACTGGACCGAGGGTAAGATTCAAAACTAACTGAATCCTTCACAACAAATCCGC
	35 Mut	AAGAACTTCTCCTCATACTAAAAGCTCTGCTCACTCTGGGATTCGTTCCTGGACCGAGCGAAAGATTCAAAACTAACTGAATCCTTCACAACAAATCCGC
	40 Mut	AAGAACTTCTCCTCATACTAAAAACTCTGCTCACTCTGGGATTCGATCCTGGACCGAGCGAACGATTCAAAACTAACTGAATCTTTTACAACAAATCCGC
	45 Mut	AAGAACTTCTCCTCATACTAAAAACTCTGCTCACTCTGGCATTATATCCTGGACCGAGCTAACGATTCAAAACTAACTGAATCTTTTACAACAAATCCCC
	50 Mut	ATGAACATCTCCTCATACTAAAAACTCTGCTCACTCTGGCATTATATCCTCAACCGAGCTAACGATTCAAAACTAACTGAATCTTTTACCACAAATCCCC
