# Supplementary material for: Highly Sensitive Virome Characterization of Aedes aegypti and Culex pipiens Complex from Central Europe and the Caribbean Reveals Potential for Interspecies Viral Transmission
Source: Pathogens. 2020 Aug 21;9(9):686. doi: 10.3390/pathogens9090686 (PMC7559857; doi:10.3390/pathogens9090686)
Supplement: Supplementary file 1 [file pathogens-09-00686-s001.zip › 2020-08-21 Supplementary files/Table_S4.pdf]

Table S4; best BLAST N hits of model sequence to NCBI database

ID	no of mutations	Total Length bp	Total Length Aligned	mean % ID	e-value	Subject length	% coverage subject	% coverage query	GI
seq1.1	10 Mut	100	100	90	1.00E-28	2121	4.7	100.0	LK931484.1
seq1.2	10 Mut	100	100	90	1.00E-28	2121	4.7	100.0	LK931484.1
seq1.3	10 Mut	100	99	91	4.00E-29	2121	4.7	99.0	LK931484.1
seq1.4	10 Mut	100	100	90	1.00E-28	2121	4.7	100	LK931484.1
seq1.5	10 Mut	100	98	91	1.00E-28	2121	4.6	98	LK931484.1
seq2.1	10 Mut	100	100	90	1.00E-28	2121	4.7	100	LK931484.1
seq2.2	10 Mut	100	99	91	4.00E-29	2121	4.7	99	LK931484.1
seq2.3	10 Mut	100	100	90	1.00E-28	2121	4.7	100	LK931484.1
seq2.4	10 Mut	100	99	91	4.00E-29	2121	4.7	99	LK931484.1
seq2.5	10 Mut	100	100	90	1.00E-28	2121	4.7	100	LK931484.1
seq1.1	15 Mut	100	91	89	1.00E-23	2121	4.3	91.0	LK931484.1
seq1.2	15 Mut	100	98	86	4.00E-22	2121	4.6	98.0	LK931484.1
seq1.3	15 Mut	100	99	86	1.00E-22	2121	4.7	99	LK931484.1
seq1.4	15 Mut	100	100	85	2.00E-21	2121	4.7	100	LK931484.1
seq1.5	15 Mut	100	98	86	4.00E-22	2121	4.6	98	LK931484.1
seq2.1	15 Mut	100	100	85	2.00E-21	2121	4.7	100	LK931484.1
seq2.2	15 Mut	100	99	86	1.00E-22	2121	4.7	99	LK931484.1
seq2.3	15 Mut	100	100	85	2.00E-21	2121	4.7	100	LK931484.1
seq2.4	15 Mut	100	99	86	1.00E-22	2121	4.7	99	LK931484.1
seq2.5	15 Mut	100	99	86	1.00E-22	2121	4.7	99	LK931484.1
seq1.1	20 Mut	100	91	85	3.00E-18	2121	4.3	91.0	LK931484.1
seq1.2	20 Mut	100	30	93	0.19	3558314	0.0	30.0	AP018533.1
seq1.3	20 Mut	100	99	81	1.00E-15	2121	4.7	99	LK931484.1
seq1.4	20 Mut	100	95	81	5.00E-15	2121	4.5	95	LK931484.1
seq1.5	20 Mut	100	98	81	5.00E-15	2121	4.6	98	LK931484.1
seq2.1	20 Mut	100	76	76	1.00E-04	2418	3.1	76	MF416388.1
seq2.2	20 Mut	100	99	81	1.00E-15	2121	4.7	99	LK931484.1
seq2.3	20 Mut	100	100	80	5.00E-15	2121	4.7	100	LK931484.1
seq2.4	20 Mut	100	99	81	1.00E-15	2121	4.7	99	LK931484.1
seq2.5	20 Mut	100	90	83	4.00E-16	2121	4.2	90	LK931484.1
seq1.1	25 Mut	100	91	79	3.00E-11	2121	4.3	91.0	LK931484.1
seq1.2	25 Mut	100	27	96	0.65	9342022	0.0	27.0	CP022221.1
seq1.3	25 Mut	100	96	77	4.00E-10	2121	4.5	96	LK931484.1
seq1.4	25 Mut	100	95	76	6.00E-08	2121	4.5	95	LK931484.1
seq1.5	25 Mut	100	31	90	2.30E+00	6257075	0.0	31	CP014258.1
seq2.1	25 Mut	100	28	93	2.30E+00	51955169	0.0	28	CP030990.1
seq2.2	25 Mut	100	99	76	5.00E-09	2121	4.7	99	LK931484.1
seq2.3	25 Mut	100	33	91	2.30E+00	1335	2.5	33	103.1
seq2.4	25 Mut	100	99	76	5.00E-09	2121	4.7	99	LK931484.1
seq2.5	25 Mut	100	82	80	1.00E-10	2121	3.9	82	LK931484.1
seq1.1	30 Mut	100	34	88	0.65	2121	1.6	34.0	746.1
seq1.2	30 Mut	100	31	90	2.3	1455109	0.0	31.0	AP010947.1
seq1.3	30 Mut	100	82	74	1.00E-03	2121	3.9	82	LK931484.1
seq1.4	30 Mut	100	34	91	5.30E-02	4979223	0.0	34	CP020872.1
seq1.5	30 Mut	100	48	81	2.30E+00	5370536	0.0	48	CP032675.1
seq2.1	30 Mut	100	31	94	5.30E-02	40226	0.1	31	JN954695.1
seq2.2	30 Mut	100	36	86	2.30E+00	35791275	0.0	36	LR537133.1
seq2.3	30 Mut	100	34	91	6.50E-01	1335	2.5	34	103.1
seq2.4	30 Mut	100	25	100	1.90E+01	3685408	0.0	25	CP002403.1
seq2.5	30 Mut	100	68	81	7.00E-07	2121	3.2	68	LK931484.1
seq1.1	35 Mut	100	25	96	7.9	2121	1.2	25.0	CP029458.1
seq1.2	35 Mut	100	30	93	2.3	163962	0.0	30.0	AC096643.2
seq1.3	35 Mut	100	36	86	2.3	58651890	0.0	36	LR697108.1
seq1.4	35 Mut	100	34	91	5.30E-02	4979223	0.0	34	CP020872.1
seq1.5	35 Mut	100	24	100	6.50E-01	2241	1.1	24	722.3
seq2.1	35 Mut	100	31	94	5.30E-02	40226	0.1	31	JN954695.1
seq2.2	35 Mut	100	31	90	2.30E+00	25217654	0.0	31	LR584086.1
seq2.3	35 Mut	100	24	100	6.50E-01	541	4.4	24	817.1
seq2.4	35 Mut	100	26	96	2.30E+00	92867	0.0	26	LN483895.1
seq2.5	35 Mut	100	26	96	2.30E+00	2639269	0.0	26	CP032100.1
seq1.1	40 Mut	100	46	85	0.19	2121	2.2	46.0	916.1
seq1.2	40 Mut	100	30	90	7.9	83001952	0.0	30.0	LR606182.1
seq1.3	40 Mut	100	33	88	2.3	2414146	0.0	33	LR025083.1
seq1.4	40 Mut	100	34	91	5.30E-02	4979223	0.0	34	CP020872.1
seq1.5	40 Mut	100	24	100	6.50E-01	2241	1.1	24	722.3
seq2.1	40 Mut	100	44	84	2.30E+00	2973626	0.0	44	CP048401.1
seq2.2	40 Mut	100	32	91	6.50E-01	25217654	0.0	32	LR584086.1
seq2.3	40 Mut	100	39	87	6.50E-01	39415425	0.0	39	LR722987.1
seq2.4	40 Mut	100	26	96	2.30E+00	92867	0.0	26	LN483895.1
seq2.5	40 Mut	100	28	93	2.30E+00	42360788	0.0	28	LR736849.1
seq1.1	45 Mut	100	30	90	7.9	2121	1.4	30.0	LR606195.1
seq1.2	45 Mut	100	23	100	2.3	3651	0.6	23.0	124.1
seq1.3	45 Mut	100	33	88	2.3	96089878	0.0	33	CP034516.1
seq1.4	45 Mut	100	28	96	1.90E-01	2187	1.3	28	206.1
seq1.5	45 Mut	100	46	80	7.90E+00	58051481	0.0	46	CP032299.1
seq2.1	45 Mut	100	41	88	5.30E-03	6343	0.6	41	171.2
seq2.2	45 Mut	100	33	88	2.30E+00	758	4.4	33	899.1
seq2.3	45 Mut	100	29	93	6.50E-01	3928141	0.0	29	CP013614.1
seq2.4	45 Mut	100	34	88	6.50E-01	3546	1.0	34	413.1
seq2.5	45 Mut	100	32	91	6.50E-01	6481972	0.0	32	CP045226.1
seq1.1	50 Mut	100	33	88	2.3	2121	1.6	33.0	322.1
seq1.2	50 Mut	100	31	94	0.65	1909907	0.0	31.0	LT594592.1
seq1.3	50 Mut	100	29	93	0.65	2464	1.2	29	886.1
seq1.4	50 Mut	100	28	96	1.90E-01	2187	1.3	28	206.1
seq1.5	50 Mut	100	36	89	6.50E-01	1563	2.3	36	379.1
seq2.1	50 Mut	100	35	89	1.90E-01	27778217	0.0	35	CP020631.1
seq2.2	50 Mut	100	40	85	2.30E+00	4305	0.9	40	949.1
seq2.3	50 Mut	100	37	86	6.50E-01	197071	0.0	37	AC244636.2
seq2.4	50 Mut	100	29	93	6.50E-01	4200651	0.0	29	CP047112.1
seq2.5	50 Mut	100	32	91	6.50E-01	6481972	0.0	32	CP045226.1
