# Supplementary material for: Highly Sensitive Virome Characterization of Aedes aegypti and Culex pipiens Complex from Central Europe and the Caribbean Reveals Potential for Interspecies Viral Transmission
Source: Pathogens. 2020 Aug 21;9(9):686. doi: 10.3390/pathogens9090686 (PMC7559857; doi:10.3390/pathogens9090686)
Supplement: Supplementary file 1 [file pathogens-09-00686-s001.zip › 2020-08-21 Supplementary files/Table_S5.pdf]

Table S5 Number of CRESS-DNA viruses hits below and above 60% query coverage

	hits to CRESS DNA viruses (%)	hits to other viruses (%)	total (%)
>60%	15 (34.9)	28 (65.1)	43 (100)
<60%	59 (64.9)	32 (35.1)	91 (100)
total	74 (55.2)	60 (44.8)	134 (100)
